# Epidemiology of Exertional Heat Illness in the Military: A Systematic Review of Observational Studies

**DOI:** 10.3390/ijerph17197037

**Published:** 2020-09-25

**Authors:** Faith O. Alele, Bunmi S. Malau-Aduli, Aduli E. O. Malau-Aduli, Melissa J. Crowe

**Affiliations:** 1 College of Healthcare Sciences, James Cook University, Townsville QLD 4811, Australia; 2 College of Medicine and Dentistry, James Cook University, Townsville QLD 4811, Australia; bunmi.malauaduli@jcu.edu.au; 3 College of Public Health, Medical and Veterinary Sciences, James Cook University, Townsville QLD 4811, Australia; aduli.malauaduli@jcu.edu.au; 4 Division of Tropical Health and Medicine, James Cook University, Townsville QLD 4811, Australia; melissa.crowe@jcu.edu.au

**Keywords:** exertional heat illness, military personnel, armed forces, risk factors, epidemiology, biomarkers

## Abstract

Exertional heat illness (EHI) is an occupational hazard among military personnel. This systematic review describes the incidence, risk factors, clinical manifestations, and biomarkers of EHI in the military. Six databases from inception to 28 May 2020 were systematically reviewed using the PRISMA guidelines. Forty-one articles met the inclusion criteria and the incidence of EHI ranged from 0.2 to 10.5 per 1000 person years, while the prevalence rates ranged from 0.3% to 9.3%. Intrinsic risk factors influencing EHI were gender, physical fitness, obesity, previous history of heat illness, and motivation, while the extrinsic factors included hot environmental conditions and service unit. Evidence suggests that loss of consciousness, absence of sweating and confusion were the common clinical features of exertional heat stroke (EHS). The mean core temperature ranged from 40 to 41.6 °C, while elevated levels of creatine phosphokinase, liver enzymes, and creatinine were common biochemical markers of EHS. The findings of the review suggest a variation in the incidence of EHI among military populations possibly due to the varying definitions used. Although some risk factors of EHI were identified, more analytical studies are needed to investigate the association between EHI and other important factors such as acclimatisation and occlusive clothing.

## 1. Introduction

Exertional heat illnesses (EHI) include a spectrum of conditions that may occur during physical exertion in hot and humid environments [1]. EHI vary in terms of classification and severity and include mild forms such as heat cramps to the more severe exertional heat stroke (EHS) [2]. The latter is considered a medical emergency and may result in profound sequelae such as multiorgan damage and death [3]. Given the morbidity and mortality associated with EHS, it is important to identify the differences between EHS and milder forms of heat-related illnesses [2]. Milder heat-related illnesses such as heat syncope and heat exhaustion are associated with a core temperature of less than 40 °C without central nervous system (CNS) disturbances [2]. On the other hand, EHS is characterised by CNS symptoms (dizziness, confusion and loss of consciousness) and an elevated core temperature of 40 °C or more [2]. 

The underpinning physiological mechanism of EHS is currently unknown, but evidence suggests that vascular endothelial damage occurs in response to hyperthermia [4]. Direct thermal injury to tissues facilitates endotoxin leakage from the intestinal mucosa and the release of cytokines into systemic circulation, thus inducing a systemic inflammatory response syndrome (SIRS) [5]. SIRS amplifies coagulation cascade, causing coagulopathies that progressively lead to disseminated intravascular coagulation (DIC), resulting in tissue damage and subsequent multiorgan dysfunction [4,5]. Multiorgan damage, renal failure, metabolic acidosis, electrolyte imbalance, acute hepatic dysfunction and death are resultant complications of EHS [6]. Given that EHS can result in damage to multiple organs, various blood biomarkers have been used to predict its severity and monitor recovery [7]. Identified biomarkers in the published literature that have been found to be elevated in response to heat shock include aspartate aminotransferase, alanine aminotransferase, lactate dehydrogenase, blood urea nitrogen, and creatine kinase [7]. 

Prior to manifestation of EHS clinical features and complications, evidence suggests that there are predisposing factors that may increase the risk of the illness in susceptible individuals [8]. Using the epidemiological triad, in 1961, Minard identified factors that contributed to the risk of heat illness and classified these into agent (climatic heat), host (related to the personnel) and environmental (related to the training centre and its program) factors [9]. In later years, Minard’s paradigm was modified, with some researchers re-classifying these risk factors into three groups—host (individual physiologically limiting), environmental and organizational (training organization) factors [10]. Individual physiological factors include poor acclimatisation to the environment, sleep deprivation, low levels of fitness and pre-existing illness [10]. Heat load at the site of activity is an environmental predisposing factor [10]. Organizational factors refer to training regulations that ensure that training schedules avoid the hottest hours of the day, adequate rehydration regimens in terms of quantity and timing, work–rest cycles and exercise intensity that matches physical fitness [10]. Other classifications that have been proposed based on Minard’s model are intrinsic (individual physiological factors) and extrinsic (related environmental and organizational) factors [8,11,12]. Minard’s paradigm is applicable to both civilian and military settings and has been used to issue safety guidelines for training officers by the United States (US) marine corps [10]. 

EHI poses an occupational hazard to the health and operational capacity of military personnel [3]. Military service personnel are at high risk of EHI when performing strenuous physical activities for extended durations in hot environments [13]. Military personnel during exertion under hot environmental conditions may produce high levels of metabolic heat, which may also be increased by the protective clothing worn [14]. As metabolic heat production increases, thermoregulatory mechanisms of heat loss are initiated. Heat dissipation or loss occurs via four physiological mechanisms—evaporation, convection, conduction and radiation [2]. During exercise in the heat, the mechanism of heat loss is via cutaneous vasodilation and evaporation of sweat [15]. As core temperature rises with continuous exertion and metabolic heat production, the physiological cooling mechanism of heat loss through evaporation of sweat may impeded by the protective clothing increasing the risk of EHI [16,17]. Furthermore, evidence suggests that high levels of motivation to push through the heat strain and beyond physiological limits without self-pacing also increases the risk of EHI [3,18].

To reduce the risk of EHI, military communities have proposed and employed extensive heat prevention guidelines and strategies [13]. These strategies include heat acclimatisation, work/rest guidelines, fluid and electrolyte replacement and identification of high-risk persons [13]. However, where there is a lack of formal prevention strategies, in particular among civilian occupational workers, there is evidence of increased risk of EHI [18]. Lucas et al. reported that the lack of formal regulated prevention measures among civilian occupations such as agricultural workers was associated with a slightly higher EHS mortality rate of 0.4/100,000 workers, compared to 0.3 per 100,000 soldiers in the US Army [18]. It is suggested that the implementation of heat management policies and plans by the US military service reduced the risk of EHI and heat exposure [18]. 

However, heat-related mortality and morbidity still occur among military personnel in spite of the preventive strategies and policies and its impact spans across individuals and the military service unit affecting the state of force readiness [8,13]. Force readiness refers to the ability to deploy to battle zones and austere environments should the need arise [8]. This may include training allied forces, insurgency work or counter terrorism, and full-scale war. Therefore, the occurrence of EHI in military service members is a threat to any military operation given that most cases of EHI are incapacitated and accounts for significant morbidity [8,17]. A study by Cox et al. revealed that at a field hospital in Camp Bastion, 1131 military admissions were reported for a total of 1368 medical patients (both civilian and military) between 2011 and 2013 [19]. A total of 612 United Kingdom (UK) military admissions were recorded, with a mean admission rate of 23.3 per 10,000 UK military per month. Heat illness was the second-leading cause of all diagnoses made in internal medicine and constituted the second largest group of UK military patients [19]. Similarly, a five-fold increase in heat stroke hospitalisation rates among US military was reported between 1980 and 2001 [13]. 

The significant impact of EHI on military operations and capacity [8] underpins the need for current research to generate robust evidence on the epidemiology of EHI in the military. While the global incidence of EHI in the military is unknown, various research studies do exist on EHI in military populations across the globe [8]. However, there is no published systematic review on the epidemiology of EHI among military service members. Therefore, to reduce the morbidity and mortality associated with EHS among military personnel and inform policies, it is important to evaluate the breadth and depth of the body of evidence. To fill this gap, this systematic review was conducted to assess available published evidence on EHI in the military. 

The specific aims of this systematic review were:

1. To summarize the incidence rate of EHI and risk factors associated with EHI in the military.
2. To characterise the clinical manifestations and biomarkers of EHI in the military.

## 2. Materials and Methods

### 2.1. Search Strategy

A literature search of multiple databases (Medline, EmCare, CINAHL, Scopus, PsycInfo, and Informit) was conducted using Preferred Reporting Items for Systematic Review and Meta-Analyses (PRISMA) guidelines from the inception of these databases to 28 May 2020 [20]. Combinations of keywords and MESH terms were used to identify articles reporting EHI in the armed forces/military in any location (Table S1).

### 2.2. Eligibility Criteria

Included in the review were peer-reviewed observational studies that reported epidemiological data on EHI, investigated at least one risk factor or included information on the biomarkers and clinical features of EHI, were published in English and included participants that were armed forces members. Studies conducted among athletes, case series, case reports, non-peer reviewed articles, commentaries, letters and conference papers were excluded from the review. 

### 2.3. Selection Strategy

**Figure 1:**
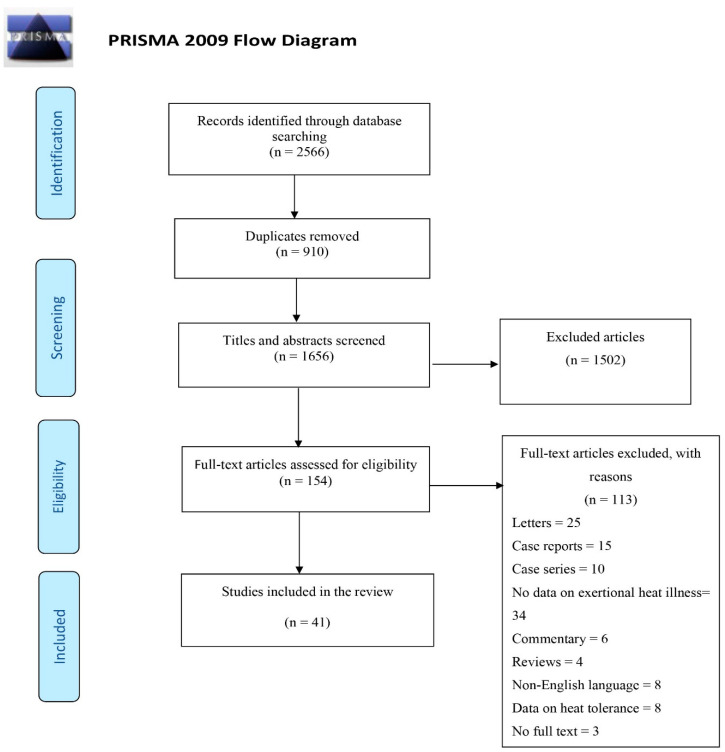
PRISMA flow chart of the systematic review selection process.

The study selection strategy is illustrated in Figure 1. F.A. conducted the search and screening of studies under the guidance of B.M.A., A.M.A. and M.C. The search and screening process were independently replicated by M.C. with discussion of any discrepancies until consensus was reached. 

### 2.4. Data Extraction

Data were extracted from the included studies by F.A. and entered into a Microsoft Excel database. The extracted data were reviewed by B.M.A. and M.C. The study designs included in the review varied and the data extracted differed depending on the study design. EHI information was extracted from observational analytical studies (cohort studies, case-control studies, case cross-over, cross-sectional studies) and descriptive cross-sectional studies. Data extracted included descriptive information on the selected studies (author, location, and year published), participants’ details (population type, age, gender and number) and study methods (design, follow-up duration and branch of the military). Other extracted information included type and definition of heat illness, incidence or prevalence of heat illness and risk factors.

### 2.5. Quality Assessment

The modified quality assessment tool for studies with diverse designs (QATSDD) critical appraisal tool was used to assess the methodological quality of the articles excluding case reports and case series [21]. The QATSDD tool is a 16-item tool that assesses the quality of both quantitative and qualitative studies. For this review, the tool was modified to exclude four items that were unrelated to the included studies. Excluded items that are related to qualitative studies were fit between stated research question and format and content of data collection tool, e.g., interview schedule (qualitative), assessment of the reliability of analytical process (qualitative only). The other excluded items were statistical assessment of the reliability and validity of measurement tool(s) (quantitative only), and evidence of user involvement in design. Each criterion in the modified QATSDD tool was awarded a minimum score of 0 and a maximum score of 3, with 0 = not at all, 1 = very slightly, 2 = moderately and 3 = complete. The total score for the methodological assessment of the included studies was 36. The scores were converted to percentages for ease of interpretation and classified as low (<50%), medium (50–80%) or high (>80%) quality of evidence. 

### 2.6. Data Synthesis and Analysis

For this review, EHI was defined as all heat-related disorders including heat stroke, heat exhaustion, heat syncope, exercise-associated muscle cramps and unspecified effects of heat and light. Data on EHI were presented as incidence and prevalence rates with calculations performed where necessary based on the available data. Most of the studies used terminologies such as exertional heat illness, exertional heat injuries, or heat illness to present the cases of EHI. A few studies reported EHI with reference to a specific diagnosis such as exertional heat stroke or heat exhaustion. 

In addition, some studies used the International Classification of Diseases to define heat illness, while others presented heat illnesses without using the ICD codes. Articles using the International Classification of Diseases defined heat stroke using the ICD diagnosis codes 992.0 (1CD 9) and T67.0 (ICD 10) and other heat illnesses were defined as heat exhaustion (992.3-5, T67 3-5) and unspecified effects of heat and light (992.9 and T67.9). Data were presented depending on the categorization of EHI. Herein, we have presented the overall prevalence and incidence of EHI where pooled data were reported irrespective of the definition used. Where heat stroke and other heat illnesses were reported separately, we have presented such data separately. In studies where prevalence rates were not reported, we calculated EHI prevalence using the total number of cases relative to the total participants or armed services population reported in the articles. Prevalence data were reported as proportions or percentages, while incidence rate data were reported as per 1000 or 100,000 person years. Efforts were made to reduce variation in the way the incidence rate data were reported. For example, studies where the denominators were reported as 100,000 or 100 were scaled to a common denominator (e.g., per 1000 person years).

Minard’s model underpinned our analysis of the risk factors of EHI [9]. We have identified and presented the risk factors and potential risk factors associated with EHI using the adapted Minard’s model [8]. Risk factors were identified where the strength of the association between exposure variables and EHI had been reported. Potential risk factors were identified based on information published in previous literature [8,9]. Risk factors reported without any measure or strength of association were classified as potential risk factors. While we have identified all risk factors (potential and real), only studies that utilised multivariate analysis were included in the synthesis. Studies that failed to control for potential confounders (without any measure or strength of association) were excluded from the synthesis and were not incorporated into the conclusion. 

Furthermore, the clinical features and blood biomarkers of EHI were identified and presented. For ease of interpretation, the number of patients in which each clinical feature was present were added together and divided by the total number of patients in the studies for which that particular feature was reported. The average values reported in the studies for the blood biomarkers were reported. Due to heterogeneity of the included studies, a meta-analysis was not conducted.

## 3. Results

**Figure 2:**
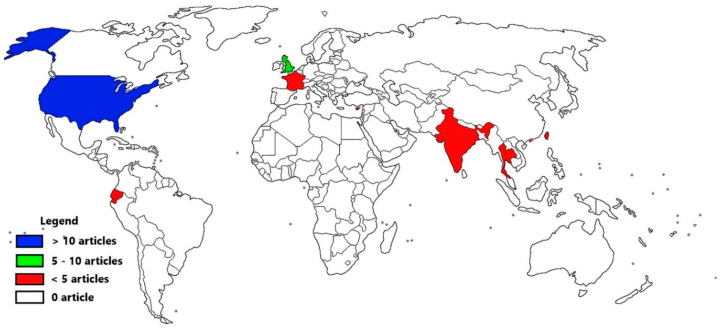
Map of the world showing the locations where studies have been conducted. Modified from Wikimedia Commons [62].

The initial search yielded a total of 2566 results, of which 910 duplicates were removed (Figure 1). The titles and abstracts were screened, and 1502 articles excluded, leaving 154 articles for full-text review. Data in Table S2 depict that 41 articles were included in the review [13,22,23,24,25,26,27,28,29,30,31,32,33,34,35,36,37,38,39,40,41,42,43,44,45,46,47,48,49,50,51,52,53,54,55,56,57,58,59,60,61], comprising 29 descriptive and cross-sectional studies [13,22,23,24,25,26,27,28,30,31,32,33,34,35,36,38,39,40,42,44,45,46,48,54,55,56,57,60,61], five cohort studies [32,49,50,51,53], six case-control studies [37,41,43,47,52,59], and one case cross-over study [58]. As shown in Figure 2, 63% (26) of the studies originated from the United States of America [13,23,24,25,26,27,28,29,30,31,32,33,34,40,41,43,45,46,49,50,53,55,58,59,60,61]; six studies from the United Kingdom [36,39,42,48,56,57]; two from Cyprus [35,39], Thailand [51,54], Taiwan [47,52], and Hong Kong [39], respectively. One article each originated from Germany [39], Singapore [37], India [38], and Ecuador [44], respectively. Approximately half of the studies (54.5%) utilised secondary data sources such as hospital registers and military databases.

### 3.1. Incidence Rate of All EHI in the Armed Forces

The overall incidence of EHI ranged from 0.2 to 10.5 per 1000 years (Table 1) in fourteen studies [13,23,24,25,26,27,28,29,30,31,37,39,55,56]. One study used a different denominator and reported the incidence of heat illness as 3.6 per 10,000 person weeks [32]. Studies that defined EHI using ICD 9 or 10 codes reported incidence rates ranging from as low as 0.2 per 1000 person years to 2.15 per 1000 person years [13,23,24,25,26,27,28,29,30,31,39]. In contrast, studies that did not use ICD codes reported incidence rates of EHI ranging from 0.76 per 1000 person years to 10.5 per 1000 person years [37,55,56]. 

**Figure 3:**
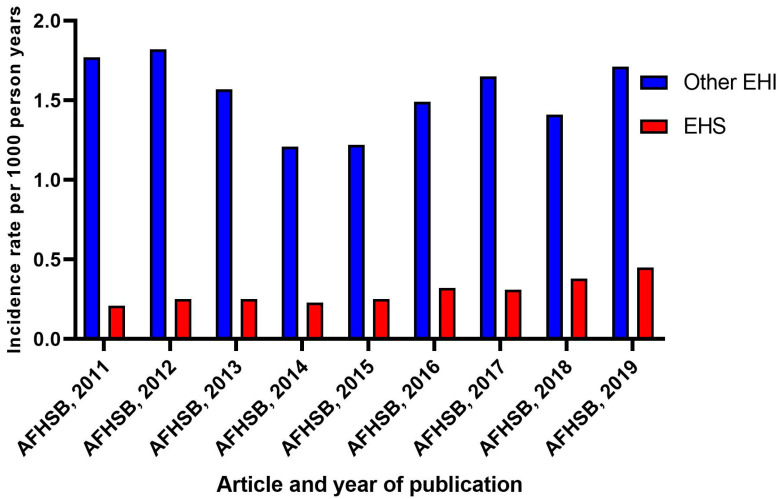
Incidence of exertional heat stroke and other exertional heat illnesses between 2011 and 2019 from the Armed Forces Health Surveillance Branch (AFHSB).

One study included a comparative analysis of the incidence data among different armed services. The findings of the study revealed that the incidence of EHI was 72.85 per 100,000 person years in the army, compared with 14.04 per 1000,000 person years in the navy and 4.85 per 100,000 person years in the air force [39]. Studies conducted by the US Armed Forces Health Surveillance Branch (AFHSB) reported heat stroke and other EHI (heat exhaustion and unspecified effects of heat and light) separately and the incidence rates varied (Figure 3). The incidence of heat stroke rose steadily from 0.21 per 100 person years in 2011 to 0.45 per person years in 2019 [23,24,25,26,27,28,29,30,31]. By contrast, the incidence rate of other EHI between 2011 and 2019 fluctuated over the 8 years. The incidence rate dropped from 1.77 per 1000 person years in 2011 to 1.21 per 1000 person years in 2015 and rose to 1.71 per 1000 person years in 2019 [23,24,25,26,27,28,29,30,31].

### 3.2. Prevalence Rate (Other Forms of Incidence Rate) of All EHI in the Armed Forces

The incidence rate based on the number of participants (prevalence rate) was reported in three studies, while for four studies, the rates of EHI were calculated to obtain the prevalence rate based on the information provided in the articles (Table 2). Overall, the prevalence rates ranged from 0.6% to 9.3% [33,34,35,44,45,50,51]. Studies published before 2010 reported prevalence rates ranging from 3.2% to 9.3% [35,44,45], while studies published from 2010 onwards reported prevalence rates ranging from as low as 0.6 to 6.6% [32,33,34,50,51]. Among studies that used the ICD 9 or 10 codes, the prevalence rates ranged from 0.6% to 1.4% [33,34,50], while the rates in studies that did not use the ICD codes ranged from 3.2% to 9.3% [35,44,45,51].

One study classified EHI events into severe and mild cases based on the complexity of the case. In this study, the prevalence of severe heat illness was 0.3% and the prevalence of mild heat illness was 1.1% [50]. Due to insufficient data, the rates of EHI in 14 studies could not be calculated using the number of participants as denominators [22,36,38,40,41,42,46,47,52,54,58,59,60,61]. 

### 3.3. Risk Factors Associated with EHI in the Military

Eighteen (18) studies reported potential risk factors that were associated with EHI (Box 1). Potential risk factors were factors reported in the included studies without any statistical analysis to determine their predictive or significant association/relationship with EHI. On the other hand, 14 studies reported the risk factors associated with EHI with statistical values (Table S3). The predictive risk factors were identified from studies where multivariate analyses have been conducted and statistical values such as odds ratio (OR), incidence density ratio (IDR), or hazard ratio (HR) with 95% confidence intervals (CI) were reported. The evidence from the 14 studies were used to synthesise the findings and draw conclusions, while the other 18 studies were not incorporated into the conclusion. Using Minard’s paradigm, the risk factors were categorized into intrinsic and extrinsic risk factors.

#### 3.3.1. Intrinsic Risk Factors

##### Sociodemographic Factors

Sociodemographic factors identified in the review were age, gender, race and marital status.

- Age

Younger age was identified as a potential risk factor in nine studies (Box 1) [23,24,25,26,27,28,29,30,31]. However, the findings from six (6) studies which assessed the relationship between EHI and age were inconsistent [33,34,49,50,53,57]. While one study reported that younger people had an increased risk [50], another study reported that older people had an increased risk of EHI [53]. In contrast, no association was reported in four studies [33,34,49,57]. There is conflicting or inconsistent evidence to show that there is a relationship between age and increased risk of EHI (Table 3 and Table S3). It should be noted that there was no consistent approach to age group comparisons across these studies.

- Gender

In nine (9) studies, gender was identified as a potential risk factor of EHI (Box 1) [23,24,25,26,27,28,29,30,31]. However, five (5) studies investigated the association between EHI and gender using multivariate analysis [13,32,49,50,53] (Table 3). The evidence suggests an increased risk of EHI among females (Table 3). Where EHI was classed into mild and severe heat illness, two studies investigated the association between gender and mild (MHI) or severe (SHI) heat illness (including heat stroke) [49,50]. One study reported that females were 2.14 times and 1.66 times more likely to develop MHI and SHI compared to their male counterparts [50]. Similarly, the other study reported that females were 1.76 times as likely as their male counterparts to develop mild heat illness (Table S3) [49]. Overall, evidence from the five studies showed that that females were more likely to experience EHI than their male counterparts with the effect size ranging from 1.18 to 2.3 [13,32,49,50,53] (Table S3).

- Race

Seven (7) studies reported race as a potential risk factor [25,26,27,28,29,30,31] (Box 1). Six studies investigated the relationship between race and EHI [13,32,33,34,41,50]. The findings were inconsistent (Table 3). Three studies found a higher risk of EHI among military members of non-white race (black) compared to white military personnel [32,41,50]. The strength of association ranged from 1.4 to 1.7 (Table S3) [32,41,50]. One study found a lower risk of EHI among non-white race (black) compared to white military personnel [13], while two studies found no association between race and EHI [33,34].

- Marital status

Another sociodemographic factor associated with risk of EHI was marital status [50]. However, there was limited evidence for the role of marital status on EHI (Table 3). Formerly married personnel were found to have a higher risk of MHI (OR 1.52; 95% CI 1.08–2.14), while unmarried personnel had a higher risk of SHI (OR 1.29 95% CI 1.05–1.59) compared to their married counterparts [50] (Table S3).

##### Physiological and Behavioural Factors

The physiological and behavioural mechanisms identified in the review were acclimatisation, motivation, sleep deprivation and hydration status.

- Acclimatisation

Heat acclimatisation is defined as an individual’s expected tolerance for a given combination of internal and external heat [57]. Lack of acclimatisation was identified in three studies as a potential risk factor [35,37,48] (Box 1). However, there is limited evidence to show that acclimatisation reduces the risk of EHI (Table 3 and Table S3). According to Stacey et al., un- acclimatised members had a lower risk of hospitalisation with EHI compared to acclimatised military personnel (OR 0.31 95% CI 0.15–0.66) [57].

- Motivation

In the military context, the desire to complete tasks and goals may be the driving force for military personnel [3]. Motivation could be measured based on the level of performance of a goal-related task which in military populations, refers to activities and intensity of exercises [63]. Three studies identified motivation as a potential intrinsic risk factor for EHI [22,36,38] with one study implying motivation based on relative exercise intensity [36]. Two studies reported an association between motivation and EHI with effect size ranging from 1.66 to 3.4 [44,57] (Table 3 and Table S3).

- Sleep deprivation

In six studies, sleep deprivation was identified as a potential risk factor [22,35,37,38,46,48] (Box 1). However, as shown in Table S3, one study reported that sleep deprivation did not increase the risk of hospitalisation for EHI (OR 0.76 95% CI 0.37–1.56) [57]. There was limited evidence to show that sleep deprivation was associated with EHI (Table 3).

- Hydration status

Four of the studies identified dehydration as a potential risk factor for EHI [22,37,42,48] (Box 1). However, there was no evidence to show that dehydration is associated with EHI (Table 3). According to Stacey et al., dehydration was not significantly associated with an increased risk for hospitalisation due to EHI (OR 1.47 95% CI 0.76–2.82) [57].

##### Anthropometric Factors

Anthropometric factors identified in this review were obesity and overweight. In this review, six studies used body mass index (BMI) as the measure to define obesity and overweight [37,41,49,50,51,59], where BMI was classified as underweight (<18.5 kgm^−2^), normal weight (18.5–25.0 kgm^−2^), overweight (25.0–29.9 kgm^−2^), and obese (≥30.0 kgm^−2^). Two other studies used a two-tiered approach to determine obesity which were BMI and percentage body fat. Participants who failed both the body fat screening and BMI assessment were classified as having excess body fat [33,34].

- Obesity and overweight

Two studies identified obesity/overweight as a potential risk factor for EHI [35,48] (Box 1). The association between obesity/overweight and EHI was investigated in eight studies [33,34,37,41,49,50,51,59] (Table S3). The evidence from the eight studies suggest that being obese or overweight increases the risk of EHI (Table 3). Common to the eight studies was the fact that the risk of EHI was higher among overweight and obese personnel with effect size ranging from 1.01 to 4.04 [33,34,37,41,49,50,51,59].

##### Fitness Factors

Physical fitness was defined using a variety of measures such as run time, step test and the US army fitness score [34,41,49,50,59]. Army fitness score (total scores for sit-ups, push-ups and runtime) was used in two studies conducted in the US army [49,50]. One study did not define the measure used to assess fitness levels [57].

- Physical Fitness

The evidence from six studies that investigated the association between physical fitness and EHI [34,41,49,50,57,59] suggests that physical fitness was associated with increased risk of EHI (Table 3). Four studies identified poor physical fitness as a potential risk factor [22,35,37,48] (Box 1). Three studies reported a non-significant association between EHI and the documented physical fitness effect size ranged from 0.84 to 1.2 [49,50,57]. Although the study by Nelson et al. identified no relationship between EHI and physical fitness using the army fitness score, the authors stated that army personnel without any documented evidence of fitness score had an increased risk of EHI (OR 2.2). The other three studies identified slower physical fitness run times and failing the step test as risk factors of EHI (Table S3), with odds ratios ranging from 1.1 to 5.61 [34,41,59].

##### Medical History Factors

The medical history factors identified in this review were a previous history of EHI, pre-existing or concurrent illness, and genetics.

- Previous heat injury or illness

Three studies identified previous heat illness as a potential risk factor [35,37,48] (Box 1). The association between EHI and a previous history of EHI was investigated in three studies (Table 3). Two studies showed a significant association between previous heat illness and EHI (Table S3) [49,50]. Nelson et al. [49] reported that a previous history of mild heat illness was significantly associated with heat stroke (HR 17.7 95% CI 8.50–36.7) [49]. Similarly, army personnel who had a prior history of heat illness were 1.77 times more likely to be at risk of severe heat illness compared to those without a history of heat illness (OR 1.77 95% CI 1.00–3.13) [50]. However, one study reported a non-significant association between previous heat illness and risk of hospitalisation with EHI (Table S3) [57]. The evidence suggests that a prior history of EHI increases the risk of another EHI episode.

- Pre-existing and concurrent illness

Eight studies identified previous or concurrent illness (such as upper respiratory tract infection, gastroenteritis/diarrhoea) as a potential risk factor of EHI [22,35,37,42,46,48,54,61] (Box 1). However, only one study investigated the relationship between concurrent illness and EHI (Table 3 and Table S3). The evidence from the study suggests that there was no association between concurrent illness and the risk of hospitalisation for EHI (OR 0.52 95% CI 0.26–1.05) [57]. Therefore, there was limited evidence to determine the association between EHI and pre-existing and concurrent illness.

- Genetics

Two of the studies identified genetic factors (such as heat shock proteins and genes involved in skeletal muscle calcium regulation, and membrane excitability) [42,61] as potential risk factors. Two studies investigated the effect of positive sickle cell trait (SCT) on EHI and the findings or evidence were conflicting or inconsistent (Table 3) [49,53]. Nelson et al. [49] reported that there was no association between SCT and either mild HI (HR 1.15 95% CI 0.84–1.56) or heat stroke (HR 1.11 95% CI 0.44–2.79) [49]. In contrast, Singer et al. [53] reported that the risk of EHI was higher among army personnel who were SCT positive compared to those who were SCT negative (HR 1.24 95% CI 1.06–1.45) [53]. Please see Table S3.

##### Medications and Lifestyle Factors

Medications, alcohol and tobacco use were identified in this review.

- Medications

In two studies, pain killers such as non-steroidal anti-inflammatory drugs (NSAIDs), antihistamines, protein supplements and ephedra-containing supplements were identified as potential risk factors of EHI [22,48] (Box 1).

However, two studies investigated the association between medications such as statins, stimulants and antipsychotics and EHI (Table 3) [49,50]. Of the two studies, one reported a significant association between MHI and antipsychotics (HR 3.25 95% CI 1.33–7.90). However, no association was observed with heat stroke [49]. The other study found a significant association between methylphenidate stimulant use and MHI but not with SHI. There was also no association between other stimulants such as amphetamines and either MHI or SHI [50]. As shown in Table S3, pain killers such as NSAIDs and opioid use were found to be significantly associated with MHI (OR 1.31 95% CI 1.05–1.64 and OR 1.92 95% CI 1.08–3.41, respectively) [50]. Overall, there is limited evidence to show that pain killers, antipsychotics and stimulants increased the risk of EHI.

- Alcohol and tobacco use

The role of alcohol in EHI was reported and identified as a potential risk factor in three studies [22,35,42] (Box 1). However, no study investigated the association between alcohol and EHI. Four studies investigated the association between tobacco use and EHI (Table 3) [34,49,50,51]. Of the four studies, three did not find any association between tobacco use or smoking and EHI [34,49,51]. However, Nelson et al. [50], reported that tobacco use was significantly associated with the risk of MHI (OR 1.55 95% CI 1.37–1.77) [50]. The evidence from the four studies suggest that there is limited evidence of no association between EHI and tobacco use (Table 3 and Table S3).

#### 3.3.2. Extrinsic Risk Factors

##### Training Factors

Training factors identified in the review were exercise intensity, clothing and equipment and service units or roles.

- Clothing and equipment

Four studies identified the use of full battle gear or thick protective clothing as a potential factor for EHI [22,37,38,46]. Only one study investigated the relationship between clothing worn and EHI (Table 3). According to Stacey et al., the risk of hospitalisation with EHI was reduced with occlusive clothing (battle gear) compared to vented clothing (Table S3) [57]. There is limited evidence to show the relationship between occlusive clothing and EHI.

- Service unit and roles

Nine studies identified service unit as a potential risk factor for EHI. The studies reported that the rate of EHI was higher among army and marine corps compared to other service units such as the air force and navy. In addition, higher rates of EHI were reported among personnel in combat roles [23,24,25,26,27,28,29,30,31]. However, six studies investigated the relationship between service units and roles and EHI (Table S3) [13,32,34,44,53,57]. One study reported a higher risk of EHI among marine corps members compared to the army (HR: 1.51 95% CI 1.22–1.88) [53]. Another study reported that national guard soldiers had a higher risk of EHI compared to soldiers in active duty (RR: 1.1 95% CI 1.0–1.2) [32]. Members in combat roles, infantry and gun crew members were reported to have a higher risk of EHI compared to service members in other roles [13,34,53]. The effect size ranged from 1.57 to 2.67 [13,34,53]. In contrast, one study reported no significant difference in EHI hospitalisation between recruits and senior rank officers [57]. Overall, the evidence suggests that there is an increased risk of EHI among service roles such as combat roles (Table 3).

##### Environmental Factors

- Hot environmental conditions and heat load

Five studies identified hot and/humid conditions as potential risk factors [22,35,37,42,46] (Box 1). However, three studies investigated the relationship between hot environmental conditions including summer season and the risk of EHI (Table 3) [50,57,58]. One of the three studies reported that the risk of EHI increased with increasing wet bulb globe temperature (WBGT) (OR: 1.11 °F¯¹ 95% CI 1.10–1.13) [58]. The next study by Nelson et al. [50] reported that during the summer season, army personnel were 22.1 (95% CI 17.3–28.2) times more likely to be at risk of MHI and 16.3 (95% CI 10.8–24.6) times more likely at risk of SHI [50]. The last study did not report any significant association between hot environmental conditions and EHI (Table S3) [57]. Overall, the evidence suggests that there is an increased risk of EHI during hot environmental conditions.

Box 1 **Potential risk factors associated with exertional heat illness.**

**Intrinsic risk factors**

**              Sociodemographic factors**
              Age [23,24,25,26,27,28,29,30,31]
              Gender [23,24,25,26,27,28,29,30,31,38]
              Race [25,26,27,28,29,30,31]
              **Physiological and behavioural factors**
              Acclimatisation [35,37,48]
              Motivation [22,36,38]
              Sleep deprivation [22,35,37,38,46,48]
              Hydration status (dehydrated) [22,37,42,48]
              **Anthropometric factors**
              Overweight/Obesity [35,48]
              **Fitness factors**
              Physical fitness [22,35,37,48]
              **Medical history factors**
              Previous heat injury [35,37,48]
              Pre-existing illness and intercurrent illness [22,35,37,42,46,48,54,61]
              Genetics [42,61]
              **Medications and lifestyle factors**
              Medications and supplements [22,48]
              Alcohol and tobacco use [22,35,42]

**Extrinsic risk factors**

              **Training factors**
              Clothing and equipment [22,37,38,46]
              Service units and roles [23,24,25,26,27,28,29,30,31]
              **Environmental factors**
              Hot environmental conditions or heat load [22,35,37,42,46]

### 3.4. Clinical Features of EHS in the Military

The clinical features of EHI (precisely, EHS) were identified from 10 studies [22,38,40,42,43,46,47,52,54,61]. The most common clinical presentations were loss of consciousness (54%), absence of sweating (47%), confusion (45%) and dehydration (44%). The average core temperature recorded in 10 studies was 40.72 °C ± 0.55, ranging from 40 to 41.6 °C. Other clinical features included nausea and vomiting, seizure, coma, headache, irrational behaviour and presence of sweating (Table 4).

### 3.5. Clinical Biomarkers of EHI in the Military

The major laboratory finding reported in the studies was a suite of biochemical markers of EHI (precisely, EHS). Other laboratory findings such as haematological findings were less frequently reported in the studies.

### 3.6. Biochemical Biomarkers

The biochemical biomarkers identified were reported as biomarkers of EHS, which is a severe type of heat illness. The most frequently reported biochemical biomarkers were elevated creatine phosphokinase (mean: 6523.1 U/L), elevated aspartate aminotransferase (mean: 180.4U/L), elevated creatinine (mean 1.89 mg/dL), elevated alanine amino transferase (mean: 166.9 U/L), and a rise in lactate dehydrogenase (mean: 575.7 U/L). Other biochemical biomarkers include metabolic acidosis, hypocalcaemia, hypophosphatemia, hyponatremia, and hypokalaemia (Table 5).

### 3.7. Haematological Biomarkers

Six studies in the review reported haematological biomarkers of EHI [38,46,47,52,54,60]. While five studies reported normal average haematocrit value ranging from 38.8% to 51.6% [38,46,47,52,54], one study reported anaemia (average haematocrit level of 37.7%) [60]. In addition, only one study identified leucocytosis (11.6 × 10^3^/µL) [60], while two studies reported average normal white blood cell counts of 10.7 × 10^3^/mcL and 12,750 cells/mm^3^, respectively [46,54].

### 3.8. Quality Assessment of the Included Studies

All the included studies were critically assessed for factors that may influence the validity of the results (Table S4). All 41 articles stated their aims and/or objectives and defined the study population or people recruited into the study. However, there were variations in reporting and/or justification of sample size. Given that all included studies were observational studies, there was an increased risk of bias. However, 14 of the included studies controlled for confounders. In addition, because heat illness is an adverse event, approximately 92% of the studies were retrospective. The data sources used were mainly secondary such as medical records or military databases (53%). The use of these data sources may be associated with bias due to inaccuracy or misclassification error (Table S4). Overall, the quality of the included studies ranged from low to high quality, with 17 studies rated as high, 13 studies rated as medium, and 11 studies as low quality (Table S4). However, the results of the assessment should be interpreted with caution. The quality assessment may be based on how the paper was written, given that some articles such as the US armed forces publications may not have reported some of the criteria listed in the appraisal tool. However, important information about EHI in military personnel was captured in these articles.

## 4. Discussion

This systematic review aimed to describe the incidence, prevalence, risk factors, clinical features and biomarkers of EHI among military personnel. The findings of this systematic review suggest that there is wide variation in the incidence and prevalence of EHI in the armed forces. Most of the risk factors associated with EHI were intrinsic factors, while the most common clinical features were neurological symptoms and biochemical markers were the key biomarkers of EHI.

### 4.1. Incidence and Prevalence of Exertional Heat Illness in the Military

The findings of this systematic review revealed wide variation in the incidence and prevalence of EHI in the armed forces. Interpretation is difficult because of the unavailability of comparable incidence data for non-military occupations. However, Dickinson et al. reported the findings of a comparative analysis in which the incidence of EHI was higher in the army, compared to the incidence among personnel in the navy and the air force [39]. In addition, the incidence varied by the type of EHI, with a higher incidence of other EHI compared to heat stroke. The wide variation in the incidence and prevalence of EHI across all included studies was attributable to the varying definitions used. Therefore, quantifying the incidence and prevalence was challenging. However, there was some evidence to show a rise in EHI incidence over the years, mainly based on the EHI data from USA. Globally, only 13 locations had published data on EHI among military personnel. Of these, a large proportion (54.5%) of the literature originated from the USA. The challenge of identifying global prevalence or incidence of EHI among military personnel has been highlighted in previous research with a large focus on the data published by the US Department of Defence [8]. The lack of data from most of the locations in the world adds to the complexity. The currently available data may therefore underestimate the true burden of EHI in the military.

### 4.2. Risk Factors Associated with EHI in the Military

Using Minard’s paradigm, many intrinsic and extrinsic risk factors were identified, but only a few were found to be predictive of EHI. Although potential risk factors were identified in some of the studies included in this systematic review, only a few risk factors were predictors of EHI (i.e., the measure of association was stated) and there was consistent evidence of their association with EHI. The intrinsic risk factors that were suggested to be associated with EHI were gender, physical fitness, obesity, motivation, and previous heat illness. Although the evidence was either limited or conflicting, other intrinsic risk factors that were found to have some form of association with EHI were age, marital status, acclimatisation, pre-existing illness, race and genetics. These intrinsic factors are in consonance with a recently published review that identified a variety of intrinsic/individual factors associated with EHI in the general population [65].

In this current study, females were more likely to experience EHI compared to their male counterparts. The higher risk of EHI among female military personnel may be due to physiological differences between males and females such as lower aerobic fitness and higher body fat in females [66,67]. The effect of other demographic variables such as age and marital status were either inconclusive or limited. The inconsistent findings of the effect of age on EHI may not be surprising given that the studied populations (military populations) are physically active and evidence suggests that age is independent of the ability to dissipate metabolic heat among aerobically fit individuals [68]. Furthermore, personnel who had never being married or were formerly married had a higher risk of EHI compared to those who were married; however, the evidence is limited. The underlying mechanism for this relationship is currently unknown and requires further exploration.

Evidence from the studies reported in this current review suggests that higher BMI and lower physical fitness are significantly associated with increased risk of EHI. This finding echoes previously documented literature that higher BMI (indicating higher body fat) increases risk of EHI [69]. In obese individuals, factors such as increased metabolic heat production during exercise, lower surface to mass ratio, and the insulating effect of subcutaneous adipose tissue may cause an increase in core body temperature and without adequate dissipation, may result in EHI [16,70,71]. In addition, evidence suggests that obese soldiers are less physically fit, as evidenced by failing the fitness test compared to non-obese soldiers [72]. However, the relationship between fitness level and obesity was not explicitly investigated in the studies in this review.

High physical/aerobic fitness has been shown to lower initial core temperature and prolong exercise time [69]. Individuals with lower aerobic fitness have shorter exercise times in hot environments than their more fit counterparts because of higher initial and final core temperatures tolerated at exhaustion [69]. In addition, the physiological adaptations occurring in response to exposure to high temperatures are similar to adaptations to aerobic training. Well-trained or fit individuals are more likely to have a larger plasma volume and may display better cardiovascular stability and central venous pressure that is similar to that of heat-acclimatised individuals [73]. However, in this review, heat acclimatisation was not significantly associated with EHI. It is important to apply caution when interpreting the association between physical fitness and EHI among military personnel, given that these findings were as a result of failing occupational fitness tests among recruits or new enlistees [65]. Furthermore, the choice of tests used to assess fitness may not provide true reflections of the aerobic fitness levels of the military personnel. According to the American Thoracic Society, the gold standard for testing cardiorespiratory fitness (aerobic fitness) is maximal oxygen uptake (VOR_2_Rmax) [74]. Therefore, more valid measures such as a VOR_2_Rmax test should, where possible, be employed in future studies to assess fitness levels. Furthermore, data that assess the association between level of fitness and EHI among long-term serving personnel are lacking.

High aerobic fitness may reduce the risk of EHI; however, motivation is a critical behavioural factor associated with EHI. In the current review, motivation was found to be an underlying intrinsic risk factor associated with EHI and its effect was documented by five studies [22,36,38,44,57]. Evidence in the literature suggests that highly trained personnel may be over-motivated and may not consider self-pacing during activities in hot conditions, increasing their risk of EHI [3]. Self-pacing refers to the reduction in work rate in response to perceived heat stress [75]. Self-pacing may be over-ridden during military activities, where battle conditions or military discipline may inhibit the ability to adjust work rate [75]. In most cases reported in the included studies, EHS occurred at the end of a forced march or military runs, highlighting a high metabolic load from attenuation of this protective mechanism [22,36,38,44,57]. The evidence from the current review buttresses the findings reported in a recent study by Gun, 2019, which stated that self-pacing was compromised during military duties in simulated battle conditions, resulting in fatal outcomes [75].

Another risk factor identified was a previous history of heat illness. Findings suggest that within the observed duration, having a prior heat illness makes military members more susceptible to heat stroke or severe heat illness [49,50]. While the data on the time lag and difference before reoccurrence of EHI are limited, documented evidence supports the finding that having a prior heat illness increases the risk of another episode of heat illness [76]. The repeated episode of heat illness may in part be due to deconditioning during recovery from EHI followed by a rapid return to activity [77]. An appropriate return to duty procedure including a heat tolerance test may be needed to determine whether an individual is fit to return to duty [77]. In addition, a gradual re-introduction of training activities may help reduce the risk of re-occurrence among individuals with a previous history of EHI [78].

Interestingly, pre-existing and concurrent illnesses such as common cold, gastrointestinal and febrile illness which have been documented in the literature to be associated with EHI were found to be a non-significant risk factor in this review [65]. However, this evidence is limited given that the predictive relationship was investigated by one study [57]. Further research is needed to elucidate the predictive role of pre-existing and concurrent illness in EHI among military personnel.

Although the evidence is less consistent, some individuals may be at increased risk of heat illness because of their ethnic backgrounds. In this review, three studies reported that non-white (black) ethnic groups were more susceptible to heat illness [32,41,50]. However, another study reported that individuals of African and Hispanic heritage had a lower risk of EHI compared to Caucasians [13]. The relationship between ethnicity and EHI is not fully understood and may need further exploration. While the association between ethnicity and heat illness is not fully understood, other factors such as genetic predisposition may play a role in the differences between ethnic groups [79].

There is a general consensus that genetic predisposition may be involved in EHI. However, there is limited data on the mode of inheritance and underlying quantitative trait loci of the specific genes responsible for EHI [42]. In this review, there were inconsistent findings on the role of genetics in EHI. The association between genetic factors such as sickle cell trait (SCT) and EHI was investigated. SCT is an inherited blood disorder and affected individuals are considered heterozygous for the sickle cell mutation in the subunit beta gene of the haemoglobin molecule [80]. Singer et al. [53] observed that SCT-positive armed force members were at increased risk of EHI compared to SCT-negative members [53]. In contrast, Nelson et al. [49] suggest that SCT may not necessarily be associated with EHI [49]. Previous evidence linked positive SCT with exercise-related adverse health outcomes (including exertional rhabdomyolysis, heat stroke and hyperthermia) in military personnel, but the biological pathway by which SCT is associated with heat illness is still unknown [53,80]. It is evident that there may be some yet to be identified underlying genetic mutations that may be responsible for EHI. More in-depth research is needed to elucidate the role of genetics in EHI.

While intrinsic factors may increase the risk of EHI, extrinsic factors such as hot environmental conditions and service unit may increase susceptibility to EHI. Evidence from the current review suggests that military personnel exercising during summer seasons and in hot weather conditions were at an increased risk of EHI [50,58]. Prolonged exposure to radiant heat with intrinsic heat production may exceed the body’s ability to dissipate heat leading to an increased risk of EHI [2]. To reduce the incidence of EHI, activity modification guidelines (AMGs) [81] were developed and, according to Wallace et al., the marine corps AMGs used WGBT indices to determine the risk of heat illness. However, majority of the EHI cases still occurred below safe green flag conditions (26.7–29.4 °C) [58]. While AMGs help to improve performance and protect personnel from EHI, evidence suggests that every branch of the US military has its own AMGs [81]. There are suggestions that future interagency collaborations are needed to improve current heat prevention policies and guidelines [81]. Nonetheless, it is imperative to note that other factors such as self-pacing and type of clothing and equipment act in synergy with hot environmental conditions to magnify the risk of EHI [2,57]. The weight of the clothing and equipment increase metabolic heat production and prevent heat loss by limiting heat exchange with the environment [81]. Although the review found no relationship between occlusive clothing and EHI, the findings need to be interpreted with caution given the limited data investigating their association. In addition, the reduced risk may have reflected the intensity of the exercise and the training guidance that advocates lower exercise intensity with additional load and insulation [57]. The underlying mechanism of restricted heat loss with the use of protective clothing underscores the reason why future studies should consider evaluating the association between EHI and occlusive clothing.

However, some military service units were more predisposed to EHI than others. Military personnel serving in combat, infantry and gun crew roles were found to have an increased risk of EHI compared to their counterparts in other service units [13,53]. Marine corps members were found to have a higher risk of EHI compared to the army [53]. These units may be required to kill enemy forces and are trained to engage in dismounted ground close combat (DGCC), among other physically arduous functions. There are multifactorial reasons for the increased risk of EHI among individuals in these high-risk groups [17]. Apart from the physically arduous functions that increase internal heat production, these individuals may need to be rapidly deployed, risking inadequate time for acclimatisation before action, which may delay heat adaptation and increase the risk of EHI [17].

### 4.3. Clinical Features of EHI in the Military

The clinical features of EHI among military personnel vary. The general symptoms include fatigue, nausea, vomiting, dehydration, and headache. More specific symptoms related to EHS identified in the current review were loss of consciousness, confusion, an elevation in body temperature above 40 °C, seizure and coma. According to current evidence, the diagnostic criteria for EHS include elevated core temperature above 40 °C and neurological manifestations such as confusion and loss of consciousness [2]. The clinical features identified in this review are similar to features of classic heat stroke. However, the presence of exertion in the military context differentiates EHI from classic heat stroke, which occurs more commonly among elderly, ambulatory individuals with co-morbidities such as diabetes, obesity and hypertension [82]. As symptoms of confusion and loss of consciousness are only associated with heat stroke and not milder forms of heat illness, then the findings of this review portray the high occurrence of EHS among military personnel. This indicates that early identification and management of these symptoms are needed given that EHS is a medical emergency and may progress to systemic inflammatory response and multiorgan system failure [11].

### 4.4. Laboratory Markers of EHS among Military Personnel

Laboratory biomarkers provide information on the evidence of multiorgan damage in EHS. Electrolyte and metabolic imbalance such as elevated CPK, elevated creatinine, hypocalcaemia, hypophosphatemia, hypokalaemia, hyponatremia and metabolic acidosis have been documented as complications of EHS [83,84]. Evidence suggests that metabolic acidosis is significantly associated with the degree of hyperthermia and is the most common acid-base change that occurs during EHI [85]. The cytotoxic effect of heat causes injured cells to leak phosphate, which binds with extracellular calcium, resulting in hypocalcaemia, while the direct effect of catecholamines or sweat losses may cause hypokalaemia [86]. With continuous exposure to heat and prolonged exertion, excessive water consumption and sweat loss may cause hyponatraemia due to excessive sodium loss [87]. Although the mechanism is not fully understood, there is some evidence to suggest that metabolic acidosis may cause an increase in urinary phosphate excretion, resulting in hypophosphatemia [88]. Another documented mechanism is hypophosphatemia, occurring as a result of glucose phosphorylation [86]. However, more evidence is needed to determine the implications of these electrolyte imbalances for EHI [60].

Elevated creatinine levels have been reported in EHS cases, an indication of acute kidney damage. The renal impairment may be related to rhabdomyolysis characterised by elevated CK levels [89]. In addition, elevated CK, alanine aminotransferase and aspartate amino transferase are indicators/predictors of liver impairment [90]. Furthermore, hepatic transferases (AST and ALT) have been documented to be elevated in heat stroke due to centrilobular necrosis following thermal injury [38]. These findings suggest that hepatic transferases could be used as surrogate markers of EHI [22]. Although the role of cytokines in EHI was not identified in this review, evidence suggests that cytokines, as immune modulators in the inflammatory process, are released during extreme hyperthermia [91]. Cytokines play key roles in mediating and attracting lymphocytes, neutrophils and monocytes to initiate and aid the healing process in damaged tissues [91]. However, in this review, only one study identified haematological biomarkers (leucocytosis, and anaemia). While the evidence in this review may be limited given the few number of articles investigating and reporting haematological biomarkers, evidence from the literature suggests that EHS results in SIRS which causes coagulopathies and DIC [4]. These biomarkers are useful in determining the severity of EHS. Therefore, having the biomarkers included as routine investigations for all cases of EHI will potentially reduce mortality and the complications associated with heat stroke [90].

### 4.5. Strengths and Limitations

To the authors’ current knowledge, this is the first systematic review that assesses the epidemiology of EHI in military populations. The review identified and quantified the burden of EHI among military personnel globally. However, most of the articles included in the review used and analysed secondary data with variabilities in these data sources, hence the need for more studies that utilise primary data. The variability in data sources may account for the varying incidence and prevalence rates reported. The use of these data sources may be associated with errors and misclassifications of the outcome of interest. These issues may have introduced misclassification bias. Another limitation was the exclusion of non-English studies, which may have resulted in missing articles on EHI in the military published in other languages. Furthermore, a large proportion of the studies were from the United States of America, and it is obvious that there is a paucity of data on EHI among military personnel globally.

### 4.6. Implications for Policy and Future Studies

The findings of this review demonstrate that there is limited information on EHI among military personnel globally. Given that EHI impacts the operational capacity of military groups, it is imperative for governments and military bodies to invest in surveillance systems that assess the health of military populations. Military leaders/commanders and supporting medical personnel should ensure that military personnel are informed regarding the clinical features and risk factors associated with EHI while enforcing countermeasures against EHI. In addition, more epidemiological studies are required globally to describe and understand the burden of EHI among military personnel. More information from military populations based in hot or hot and humid regions would be beneficial. In addition, climate change is warming the Earth’s surface, the resultant increasing frequency and severity of extreme weather events including increased temperature will exacerbate hazards such as EHI which pose a significant risk/threat to the operational capability of military services globally [92]. Given that EHIs are preventable, the aim of military groups should be primary prevention, which involves early identification of the risk factors as well as initiation of strategies to mitigate the risk and warning strategies to reduce heat stress conditions. In addition, with increasing frequency and intensity of heat waves, current AMGs need to be reviewed and revised to ensure the safety of military personnel under harsh environmental conditions [81].

In this review, we focused on the predictors of EHI. While we uncovered several risk factors (intrinsic and extrinsic), there was limited information on the predictive role of some of these factors in EHI. For example, it may be considered as common knowledge that acclimatisation, concurrent illness and occlusive clothing play a role in EHI. However, among military personnel, very few studies have investigated and identified the predictive role of motivation, acclimatisation, concurrent or pre-existing illness and occlusive clothing. Therefore, it is important that future studies on EHI in the military should consider investigating the predictive role of all potential risk factors. This will provide a better understanding of the role of these factors in the development of EHI among military personnel and allow for more effective planning to reduce the incidence of EHI.

Furthermore, while there are plausible reasons for the electrolyte imbalance that occurs during EHI, it is important to note that the association between heat illness and some biomarkers remains unclear and needs further investigation. Future studies should consider identifying genetic biomarkers that could serve as surrogate markers for the early detection of EHI in individuals at risk and to reduce the long-term incidence of EHI. These biomarkers could prove to be a useful set of tools during screening exercises of potential defence force recruits at the point of entry into the armed forces.

## 5. Conclusions

EHI impacts on the health and operational capability of military personnel. The incidence and prevalence of EHI varied in this review. However, the evidence suggests that the main risk factors associated with EHI were gender, physical fitness, obesity, previous history of heat illness, motivation, hot environmental conditions, and service unit. More epidemiological studies are required globally to quantify the burden of EHI and to identify under-investigated risk factors as well as the clinical features of EHI in military populations.

## Figures and Tables

**Table 1:** Incidence of all exertional heat illnesses in the military.

Author and Year	Location	Year of Study	Study Design	Participants and Branch of the Military	Overall Incidence of Exertional Heat Illness
Dickson, 1994 [39]	UK	1981–1991	Cross-sectional	326,500 UK tri-service members	0.40/1000 person years *
	Germany			(royal navy, royal air force, and the army) deployed to the different locations	
	Hong Kong				
	Cyprus				
	Gibraltar				
Chung and Pin, 1996 [37]	Singapore	1992–1994	Case-control	218 Singapore soldiers with heat disorders and 537 controls	1992: 8.1/1000 person years
					1993: 7.0/1000 person years
					1994: 10.5/1000 person years
Smalley et al., 2003 [55]	USA	1999	Cross-sectional	US air force members (51 cases)	1.3/1000 person years
Carter et al., 2005 [13]	USA	1980–2002	Cross-sectional	5246 US army soldiers	1980: 0.2/1000 person years *
					1991: 0.55/1000 person years *
					2002: 0.2/1000 person years *
Armed Forces Health Surveillance Branch, 2011 [30]	USA	2010	Descriptive cross-sectional	US armed forces, 2887 cases of exertional heat illness	1.98/1000 person years
Armed Forces Health Surveillance Branch, 2012 [23]	USA	2011	Descriptive cross-sectional	US armed forces, 3014 cases of exertional heat illness	2.07/1000 person years
Armed Forces Health Surveillance Branch, 2013 [24]	USA	2012	Descriptive cross-sectional	US armed forces, 2622 cases of exertional heat illness	1.82/1000 person years
Armed Forces Health Surveillance Branch, 2014 [31]	USA	2013	Descriptive cross-sectional	US armed forces, 2025 cases of exertional heat illness	1.44/1000 person years
Armed Forces Health Surveillance Branch, 2015 [25]	USA	2014	Descriptive cross-sectional	US armed forces, 2027 cases of exertional heat illness	1.47/1000 person years
Armed Forces Health Surveillance Branch, 2016 [26]	USA	2015	Descriptive cross-sectional	US armed forces, 2344 cases of exertional heat illness	1.81/1000 person years
Stacey et al., 2016 [56]	UK	2009–2013	Cross-sectional	UK army; 565 cases of heat illness	0.76/1000 person year *
Armed Forces Health Surveillance Branch, 2017 [27]	USA	2016	Descriptive cross-sectional	US armed forces, 2536 cases of exertional heat illness	1.96/1000 person years
Armed Forces Health Surveillance Branch, 2018 [28]	USA	2017	Descriptive cross-sectional	US armed forces, 2163 cases of exertional heat illness	1.79/1000 person years
Armed Forces Health Surveillance Branch, 2019 [29]	USA	2018	Descriptive cross-sectional	US armed forces; 2792 cases of exertional heat illness	2.15/1000 person years
Barnes et al., 2019 [32]	USA	2014–2018	Retrospective cohort study	352,739 US army soldiers	3.6/10,000 BCT person weeks

* Incidence rates were converted to per 1000 person year; UK: United Kingdom; USA: United States of America.

**Table 2:** Prevalence of all exertional heat illness in the armed forces.

Author and Year	Location	Year of Study	Study Design	Participants and Branch of the Military	Overall Prevalence of Exertional Heat Illness (%)
Kerstein et al., 1984 [45]	United States of America	Not stated	Cross-sectional	6010 Marines	4.8 *
Harris et al., 1985 [44]	Ecuador	1982	Cross-sectional using hospital records	216 Naval cadets	9.3
Bricknell 1994 [35]	Cyprus	1990–1994	Cross-sectional	3000 British (UK) soldiers	3.2
Bedno et al., 2010 [33]	United States of America	Feb 2005–Sept 2006	Cross-sectional	9967 US army soldiers	0.6 *
Bedno et al., 2014 [34]	United States of America	Feb 2005–Sept 2006	Cross-sectional	9455 US army soldiers	0.7 *
Nelson et al., 2018 [50]	United States of America	2011–2014	Retrospective cohort study	238,168 US army soldiers	1.4 *
Nutong et al., 2018 [51]	Thailand	May–July 2013	Cohort study	809 Royal Thai army soldiers (newly inducted conscripts)	6.6

* The prevalence was calculated based on number of heat illness cases and the total number of participants reported in the article; UK: United Kingdom; US: United States of America.

**Table 3:** Risk factors associated with exertional heat illnesses.

Risk Factor	Positive Association/Increased the Risk of EHI	Negative Association/Decreased the Risk of EHI	No Association with EHI	Comment
**Intrinsic**				
**Sociodemographic characteristics**				
**Age**				Inconsistent or conflicting evidence
Older age	Singer et al., 2018 [53]		Bedno et al., 2010 [33]	
			Bedno et al., 2014 [34]	
			Stacey et al., 2015 [57]	
			Nelson et al., 2018 [49]	
Younger age	Nelson et al., 2018 [50]			
Female gender	Carter et al., 2005 [13]			Suggested evidence of increased risk of EHI
	Nelson et al., 2018 [49]			
	Nelson et al., 2018 [50]			
	Singer et al., 2018 [53]			
	Barnes et al., 2019 [32]			
Marital status (never/formally married)	Nelson et al., 2018 [50]			Limited evidence of increased risk of EHI
Race/ethnicity	Gardner et al., 1996 [41]			Suggested evidence of increased risk of EHI
Non-whites vs. whites	Nelson et al., 2018 [50]	Carter et al., 2005 [13]	Bedno et al., 2010 [33]	
	Barnes et al., 2019 [32]		Bedno et al., 2014 [34]	
**Physiological and behavioural factors**				
Acclimatisation		Stacey et al., 2015 [57]		Limited evidence of decreased risk
Motivation				Suggested evidence of increased risk of EHI
*Self-pacing* vs. *group pacing*	Stacey et al., 2015 [57]			
*Exercise intensity*	Harris et al., 1985 [44]			
Sleep deprivation			Stacey et al., 2015 [57]	Limited evidence of no risk
Hydration status			Stacey et al., 2015 [57]	Limited evidence of no risk
**Anthropometric factors**				
Overweight/Obesity/High BMI	Chung and Pin 1996 [37]			Suggested evidence of increased risk of EHI
	Gardener et al., 1996 [41]			
	Wallace et al., 2006 [59]			
	Bedno et al., 2010 [33]			
	Bedno et al., 2014 [34]			
	Nelson et al., 2018 [49]			
	Nelson et al., 2018 [50]			
	Nutong et al., 2018 [51]			
**Fitness factors**				
Physical fitness	Gardener et al., 1996 [41]		Stacey et al., 2015 [57]	Suggested evidence of increased risk of EHI#
	Wallace et al., 2006 [59]		Nelson et al., 2018 [49]	
	Bedno et al., 2014 [34]			
	Nelson et al., 2018 [50] *		Nelson et al., 2018 [50] *	
**Medical history**				
Previous HI	Nelson et al., 2017 [49]		Stacey et al., 2015 [57]	Suggested evidence of increased risk of EHI
	Nelson et al., 2018 [50]			
Pre-existing illness			Stacey et al., 2015 [57]	Limited evidence of decreased risk
Genetics (SCT)	Singer et al., 2018 [53]		Nelson et al., 2017 [49]	Inconsistent or conflicting evidence
**Medications and Lifestyle factors**				
Pain killers (NSAIDs and opioids)	Nelson et al., 2018 [50]			Limited evidence of increased risk
Stimulants	Nelson et al., 2018 [50]		Nelson et al., 2017 [49]	Inconsistent or conflicting evidence
Antipsychotics	Nelson et al., 2017 [49]			Limited evidence of increased risk
Statins			Nelson et al., 2017 [49]	Limited evidence of no risk
Tobacco smoking	Nelson et al., 2018 [50]		Bedno et al., 2014 [34]	Limited/no evidence of the risk
			Nelson et al., 2017 [49]	
			Nutong et al., 2018 [51]	
**Extrinsic**				
**Training factors**				
Clothing (occlusive)		Stacey et al., 2015 [57]		Limited evidence of decreased risk
Service units and roles	Harris et al., 1985 [44]	Stacey et al., 2015 [57]		Suggested evidence of increased risk of EHI
	Carter et al., 2005 [13]			
	Bedno et al., 2014 [34]			
	Barnes et al., 2019 [32]			
**Environmental factors**				
Hot environmental conditions	Wallace et al., 2005 [58]		Stacey et al., 2015 [57]	Suggested evidence of increased risk of EHI
	Nelson et al., 2018 [48]			

* The study stated no association between the army fitness score and EHI but also identified that army personnel without a documented fitness score had an increased risk of EHI.

**Table 4:** Clinical manifestations of exertional heat stroke in the military.

Clinical Manifestation	n/N *	%
Unconsciousness [22,54]	116/214	54
Absence of sweating [38]	37/78	47
Confusion or disorientation [22,38,61]	126/274	45
Dehydration [38]	34/78	44
Nausea and vomiting [22,38]	51/267	19
Coma [22,38]	52/308	17
Seizures [22,38,54]	48/292	16
Presence of profuse sweating [22]	30/189	16
Fatigue [22]	25/189	13
Violent or irrational behaviour [22,38]	29/267	11
Headache [22]	8/189	4
	**Mean**	**SD**
Core temperature [22,38,40,42,43,46,47,52,54,61] ^‡^	40.72 °C	0.55

* n is the number of patients with the reported clinical feature in all the studies; N is the total number of patients in the studies; ^‡^ mean core temperature.

**Table 5:** Biochemical markers of Exertional Heat Stroke in the military.

Biochemical Markers	Average Values ^‡^	Range	Normative Values (Unit of Measurement)
Creatine phosphokinase (CPK) [22,38,40,42,43,46,47,54,60,61]	6523.1	1251–27985.6	22–26 (U/L) ^#^
Aspartate aminotransferase (AST) [22,38,40,43,46,52,54,60,61]	180.4	92.9–204.25	10–40 (U/L) ^#^
Creatinine [40,43,46,47,52,54,60]	1.89	1.4–1.96	0.6–1.3 (mg/dL) ^^^
Alanine aminotransferase (ALT) [22,38,52,54,60,61]	166.9	90.8–402	6–43 (U/L) ^#^
Lactose dehydrogenase (LDH) [38,43,46,54,61]	575.7	387.1–794.8	140–280 (U/L) ^^^
Bicarbonate [43,47,54]	18.9 *	18.2–19.7 *	21–29 (mmol/L) ^†^
Anion gap [46] *	24.3 *	NS	10–20 (mEq/L)
Calcium [47,54,60]	8.3	8.2–8.4	8.6–10.6 (mg/dL) ^†^
Phosphate [38,54]	0.85	0.8–8.89	0.8–1.5 (mmol/L) ^†^
Sodium (Na^+^) [38] *	124 *	103–140 *	133–146 (mmol/L) ^†^
Potassium (K^+^) [38] *	3.45 *	1.8–4.8 *	3.5–5.3 (mmol/L) ^†^

^‡^ Average of the values reported by the different studies; * Reported only by one study and values stated as reported in the study. NS: not stated. ^#^ Reference range for [61] was used; ^^^ reference range for [47] was used. ^†^ Reference range obtained from [64]. Note that ranges may vary slightly across the different studies.

## Supplementary Materials

The following are available online at https://www.mdpi.com/1660-4601/17/19/7037/s1, Table S1: Medline search strategy; Table S2: Summary of all included studies (presented by year of publication from oldest to most recent); Table S3: Characteristics of the risk factors associated with EHI, supported by the effect sizes (strength of association); Table S4: Quality assessment of included studies using the modified quality assessment tool for studies with diverse designs (QATSDD).
