# Supplementary material for: Epidemiology of Exertional Heat Illness in the Military: A Systematic Review of Observational Studies
Source: Int J Environ Res Public Health. 2020 Sep 25;17(19):7037. doi: 10.3390/ijerph17197037 (PMC7579124; doi:10.3390/ijerph17197037)
Supplement: Supplementary file 1 [file ijerph-17-07037-s001.zip › ijerph-927752-Supplementary/Supplementary Table 1.docx]

**Table S1: Medline search strategy**

|  | **Search terms ad combinations** |
| --- | --- |
| **1** | "heat strok*" OR "heatstrok*" OR "heat collapse" OR "heat exhaustion" OR "heat prostration" OR "heat cramp" OR "heat cramps" OR "heat stress disorder" OR "heat stress disorders" OR "heat stress syndrome" OR "heat stress syndromes" OR "thermal stress" OR "heat illness" OR "heat illnesses" OR "heat injury" OR "heat injuries" OR "heat disorder" OR "heat disorders" OR "heat related diseases" OR "heat related disorder" OR "heat related disorders" OR "heat related illness" OR "heat related illnesses" OR "heat related injuries" OR "heat related injury" OR "environmental heat illness" OR "heat stress" |
| **2** | temperature OR “hot weather” OR heat OR “hot temperature” OR “hot temperatures” OR humidity OR humidities OR “relative humidity” OR activit* OR “strenuous work” OR “physical effort” OR “physical efforts” OR “physical exertion” OR “physical exertions” OR “protective clothing” OR clothing OR “personal protective equipment” OR “water consumption” OR drinking OR “water intake” OR “alcohol drinking” OR “alcohol consumption” OR caffeine OR “caffeine consumption” OR medication OR medications OR drugs OR “pharmaceutic preparations” OR "pharmaceutical preparations” OR “pharmaceutical products” OR pharmaceuticals OR acclimatization OR acclimation OR “physical fitness” OR obesity OR overweight OR “human genetics” OR “medical genetics” OR ethnicity OR “ethnic group” OR “ethnic groups” OR ethnicity OR nationality |
| **3** | “air force personnel” OR “armed forces personnel” OR “army personnel” OR “coast guard” OR marines OR military OR “military deployment” OR “military personnel” OR “navy personnel” OR sailor OR sailors OR soldier OR soldiers OR submariner OR submariners |
| **4** | 1 AND 2 AND 3 |
