# Supplementary material for: Epidemiology of Exertional Heat Illness in the Military: A Systematic Review of Observational Studies
Source: Int J Environ Res Public Health. 2020 Sep 25;17(19):7037. doi: 10.3390/ijerph17197037 (PMC7579124; doi:10.3390/ijerph17197037)
Supplement: Supplementary file 1 [file ijerph-17-07037-s001.zip › ijerph-927752-Supplementary/Supplementary Table 2.docx]

**Table S2 Summary of all included studies (presented by year of publication from oldest to most recent)**

| **Author and year** | **Location** | **Year of study** | **Study design** | **Study participants** | **Study findings* (*Incidence rate and prevalence rate for EHI)** | **OR/RR/HR/IDR (95% CI)** |
| --- | --- | --- | --- | --- | --- | --- |
| Kerstein *et al*, 1984[39] | United States of America | NS | Cross-sectional | 6010 Marines | Prevalence rate: 4.8% |  |
| Harris *et al*, 1985[38] | Ecuador | 1982 | Cross-sectional | 216 Naval cadets | **Extrinsic risk factors** |  |
|  |  |  |  |  | Running the extra kilometre: | RR 3.4 (1.3 – 9.0) |
|  |  |  |  |  | First year cadets than in other cadets: | RR: 3.0 (1.3 – 7.0) |
| Bricknell, 1994[29] | Cyprus | 1990 - 1994 | Cross-sectional | 3000 British soldiers | Prevalence rate: 3.2 |  |
|  |  |  |  |  | **Potential intrinsic risk factors** |  |
|  |  |  |  |  | Lack of acclimatization |  |
|  |  |  |  |  | Alcohol |  |
|  |  |  |  |  | Pre-existing illness |  |
|  |  |  |  |  | Previous heat injury |  |
|  |  |  |  |  | Recent vaccination |  |
|  |  |  |  |  | Obesity |  |
|  |  |  |  |  | Sleep deprivation |  |
|  |  |  |  |  | Low physical fitness |  |
| Dickson, 1994[33] | United Kingdom | 1981 - 1991 | Cross-sectional | 326,500 Tri service members | Incidence rate: 0.40/1000 person-years* |  |
|  | Germany |  |  | (Royal Navy, Royal Airforce, and the Army) |  |  |
|  | Hong Kong |  |  |  |  |  |
|  | Cyprus |  |  |  |  |  |
|  | Gibraltar |  |  |  |  |  |
| Shieh *et al*, 1995[46] | Taiwan | NS | Case-control | 12 military recruits | **Biochemical blood markers** |  |
|  |  |  |  |  | Elevated AST |  |
|  |  |  |  |  | Elevated ALT |  |
|  |  |  |  |  | Elevated Creatinine |  |
| Bricknell, 1996[30] | Cyprus | 1986 - 1994 | Cross sectional (Descriptive) | UK soldiers | **Heat illness casualties** |  |
|  |  | 1986 - 1994 |  |  | UK land forces: 490 (cases) |  |
|  |  | 1990 - 1994 |  |  | Cyprus: 97 (cases) |  |
| Chung and Pin, 1996[31] | Singapore | 1992 - 1994 | Case - control | 218 soldiers | **Incidence rate**:  1992: 8.1/1000 person-years |  |
|  |  |  |  |  | 1993: 7.0/1000 person-years |  |
|  |  |  |  |  | 1994: 10.5/ 1000 person-years |  |
|  |  |  |  |  | **Intrinsic risk factor** |  |
|  |  |  |  |  | Obesity (BMI < 27 Kgm^-2^ vs BMI > 27 Kgm^-2^): | OR 4.3(NS) |
| Gardner *et al,* 1996[35] | United states of America | 1988 - 1992 | Case-control | Marine corps | **Intrinsic risk factors** |  |
|  |  |  |  | 391 cases | Non-whites vs whites |  |
|  |  |  |  | 1467 controls | **BMI** |  |
|  |  |  |  |  | BMI 22 Kgm^-2^ - <26 vs <22 Kgm^-2^ | OR 1.9 (NS) |
|  |  |  |  |  | BMI >=26 Kgm^-2^ vs <22 Kgm^-2^ | OR 1.6 (NS) |
|  |  |  |  |  | **1.5-mile physical fitness test** |  |
|  |  |  |  |  | Run time of 10 mins - < 12 mins vs < 10 mins | OR 1.1(NS) |
|  |  |  |  |  | Run time of >= 12 mins vs < 10mins | OR 3.4 (NS) |
|  |  |  |  |  | **3-mile physical fitness test** |  |
|  |  |  |  |  | Run time of 20 mins - < 23 mins vs < 20 mins | OR 2.1 (NS) |
|  |  |  |  |  | Run time of >= 23 mins vs < 20 mins | OR 4.2 (NS) |
| Lin *et al*, 2003[41] | Taiwan | 1999-2001 | Case-control | 12 military recruits | **Biochemical blood marker** |  |
|  |  |  |  |  | Elevated blood urea nitrogen |  |
|  |  |  |  |  | Elevated creatinine |  |
|  |  |  |  |  | Elevated creatine phosphokinase |  |
| Smalley *et al*, 2003[49] | United States of America | 1999 | Cross-sectional | US Airforce recruits (51 cases) | Incidence rate: 1.3/ 1000 person-years |  |
|  |  |  |  |  |  |  |
| Hakre *et al*, 2004[37] | United States of America | 1988– 1992 | Case -control | Marine Corps recruits | **Intrinsic risk factor** |  |
|  |  |  |  | 61 cases | Older age (≥23years vs 17 -18 years): | OR 2.8 (1.2 – 6.5) |
|  |  |  |  | 504 controls |  |  |
|  |  |  |  |  | **Biochemical blood markers** |  |
|  |  |  |  |  | Elevated BUN |  |
|  |  |  |  |  | Elevated creatinine |  |
|  |  |  |  |  | Hypernatremia |  |
|  |  |  |  |  | Hyperkalaemia |  |
|  |  |  |  |  | Metabolic acidosis |  |
|  |  |  |  |  | Hypoglycaemia |  |
|  |  |  |  |  | Elevated Creatinine Phosphokinase |  |
|  |  |  |  |  | Elevated Uric acid |  |
|  |  |  |  |  | Elevated AST |  |
|  |  |  |  |  | Elevated LDH |  |
| Sonna *et al*, 2004[55] | United States of America | NS | Cross-sectional (Descriptive) | 7 soldiers | **Potential intrinsic risk factors** |  |
|  |  |  |  |  | Pre-existing illness |  |
|  |  |  |  |  | Genetics |  |
|  |  |  |  |  | **Clinical features** |  |
|  |  |  |  |  | Malaise |  |
|  |  |  |  |  | Confusion |  |
|  |  |  |  |  | Fatigue |  |
|  |  |  |  |  | Nausea |  |
|  |  |  |  |  | **Biochemical markers** |  |
|  |  |  |  |  | Elevated alanine aminotransferase |  |
|  |  |  |  |  | Elevated aspartate aminotransferase |  |
|  |  |  |  |  | Elevated creatine phosphokinase |  |
|  |  |  |  |  | Elevated lactate dehydrogenase |  |
|  |  |  |  |  | Elevated creatinine |  |
|  |  |  |  |  | **Haematological blood markers** |  |
|  |  |  |  |  | Leucocytosis |  |
| Carter et al, 2005[13] | United States of America | 1980 - 2002 | Cross-sectional | 5246 US Army soldiers | 1980: 0.2/1000 person-years* |  |
|  |  |  |  |  | 1991: 0.55/1000 person-years* |  |
|  |  |  |  |  | 2002: 0.2/ 1000 person-years* |  |
|  |  |  |  |  | **Intrinsic risk factors** |  |
|  |  |  |  |  | Female | IDR 1.18 (1.09 – 1.27) |
|  |  |  |  |  | African and Hispanic vs Caucasians | IDR 0.76 (0.71 – 0.82) |
|  |  |  |  |  | **Extrinsic risk factors** |  |
|  |  |  |  |  | Infantry and gun crew | 1DR 2.67 (1.71 – 2.89) |
|  |  |  |  |  | Recruits from Northern states vs Southern states | IDR 1.69 (1.42 – 1.90) |
| Wallace *et al*, 2005[52] | United States of America | 1979 - 1997 | Case - crossover | 2069 Marine Corps recruits | **Extrinsic risk factors** |  |
|  |  |  |  |  | Increasing WGBT | OR: 1.11 °F¯¹ (1.10 - 1.13) |
|  |  |  |  |  | WBGT at time of event | OR: 1.10 °F¯¹ (1.08 - 1.11) |
|  |  |  |  |  | Average WBGT on the previous day | OR: 1.03 °F¯¹ (1.02 - 1.05) |
| Wallace *et al,* 2006[53] | United States of America | 1986 - 1996 | Case - control | Marine Corps | **Intrinsic risk factors** |  |
|  |  |  |  | 627 cases and 1802 controls | **Women** |  |
|  |  |  |  |  | Run time of >= 6.9 mins vs < 5.8 mins | OR 5.30 (1.59 - 17.64) |
|  |  |  |  |  | **Men** |  |
|  |  |  |  |  | BMI 22 Kgm^-2^ - <26 Kgm^-2^ VS <22 Kgm^-2^ | OR 1.51 (1.17 - 1.93) |
|  |  |  |  |  | BMI >=26 Kgm^-2^ VS <22 Kgm^-2^ | OR 2.10 (1.59 - 2.78) |
|  |  |  |  |  | Run time of 10.3 - < 11.7 mins vs < 10.3 mins | OR 1.51 (1.10 - 2.06) |
|  |  |  |  |  | Run time of 11.7 - < 12.9 vs <10.3mins | OR 3.62 (2.61 - 5.03) |
|  |  |  |  |  | Run time of >= 12.9 mins vs < 10.3 mins | OR 5.61 (3.73 - 8.45) |
| Sithinamsuwan *et al*, 2009[48] | Thailand | 1995 - 2007 | Cross-sectional | 25 Soldiers | **Predisposing intrinsic risk factors** |  |
|  |  |  |  |  | Pre-existing illness |  |
|  |  |  |  |  | **Clinical features** |  |
|  |  |  |  |  | Fever |  |
|  |  |  |  |  | Core temperature: 41.6℃ |  |
|  |  |  |  |  | Altered consciousness |  |
|  |  |  |  |  | Seizure |  |
|  |  |  |  |  | **Biochemical markers** |  |
|  |  |  |  |  | Elevated blood urea nitrogen |  |
|  |  |  |  |  | Elevated creatinine |  |
|  |  |  |  |  | Elevated lactate dehydrogenase |  |
|  |  |  |  |  | Elevated aspartate aminotransferase |  |
|  |  |  |  |  | Elevated alanine aminotransferase |  |
|  |  |  |  |  | Elevated creatine phosphokinase |  |
|  |  |  |  |  | Hypokalaemia |  |
|  |  |  |  |  | Hyponatremia |  |
| Bedno *et al,* 2010 [27] | United States of America | 2005 - 2006 | Cross-sectional | 9667 Male US Army recruits | Prevalence rate: 0.6% |  |
|  |  |  |  |  | **Intrinsic risk factors** |  |
|  |  |  |  |  | Excess body fat vs no excess body fat | OR: 3.63 (1.92 - 6.85) |
|  |  |  |  |  | Age ≥ 20 years vs < 20 years | OR: 0.91 (0.52 – 1.58) |
|  |  |  |  |  | Black vs white and other | OR: 0.75 (0.27 – 2.10) |
| Armed Forces Health Surveillance Branch, 2011[24] | United States of America | 2010 | Cross-sectional (Descriptive) | US Armed Forces (311 cases of heat stroke and 2576 of other heat injury) | Incidence rate: 1.98/1000 person-years |  |
|  |  |  |  |  | **Potential intrinsic risk factor** |  |
|  |  |  |  |  | Younger age |  |
|  |  |  |  |  | Non-white ethnicity |  |
|  |  |  |  |  | **Potential extrinsic risk factors** |  |
|  |  |  |  |  | Combat specific occupations |  |
|  |  |  |  |  | Army and Marine Corps members |  |
| Armed Forces Health Surveillance Branch, 2012[17] | United States of America | 2011 | Cross-sectional (Descriptive) | US Armed Forces (362 cases of heat stroke and 2652 of other heat injury) | Incidence rate: 2.07/1000 person-years |  |
|  |  |  |  |  | **Potential intrinsic risk factor** |  |
|  |  |  |  |  | Younger age |  |
|  |  |  |  |  | **Potential extrinsic risk factors** |  |
|  |  |  |  |  | Combat specific occupations |  |
|  |  |  |  |  | Army and Marine Corps members |  |
| Armed Forces Health Surveillance Branch, 2013[18] | United States of America | 2012 | Cross-sectional (Descriptive) | US Armed Forces (365 cases of heat stroke and 2257 of other heat injury) | Incidence rate: 1.82/1000 person-years |  |
|  |  |  |  |  | **Potential intrinsic risk factors** |  |
|  |  |  |  |  | Younger age |  |
|  |  |  |  |  | **Potential extrinsic risk factors** |  |
|  |  |  |  |  | Combat specific occupations |  |
|  |  |  |  |  | Army and Marine Corps members |  |
| Armed Forces Health Surveillance Branch, 2014[25] | United States of America | 2013 | Cross-sectional (Descriptive) | US Armed Forces (324 cases of heat stroke and 1701 of other heat injury) | Incidence rate: 1.44/1000 person-years |  |
|  |  |  |  |  | **Potential intrinsic risk factors** |  |
|  |  |  |  |  | Younger age |  |
|  |  |  |  |  | Asian/Pacific Islanders |  |
|  |  |  |  |  | **Potential extrinsic risk factors** |  |
|  |  |  |  |  | Combat specific occupations |  |
|  |  |  |  |  | Army and Marine Corps members |  |
| Abriat *et al*, 2014[16] | France | 2004 - 2006 | Cross-sectional (Descriptive) | French Army (182 cases of EHI) | **Potential intrinsic risk factors** |  |
|  |  |  |  |  | Motivation |  |
|  |  |  |  |  | Dehydration |  |
|  |  |  |  |  | Sleep deprivation |  |
|  |  |  |  |  | Pre-existing illness |  |
|  |  |  |  |  | Alcohol |  |
|  |  |  |  |  | Low fitness levels |  |
|  |  |  |  |  | Protein supplements |  |
|  |  |  |  |  | **Potential extrinsic risk factors** |  |
|  |  |  |  |  | Exercise intensity |  |
|  |  |  |  |  | Clothing and equipment |  |
|  |  |  |  |  | Ambient temperature and relative humidity |  |
|  |  |  |  |  | **Clinical features** |  |
|  |  |  |  |  | Unconsciousness |  |
|  |  |  |  |  | Confusion |  |
|  |  |  |  |  | Vomiting |  |
|  |  |  |  |  | Intense fatigue |  |
|  |  |  |  |  | Ataxia |  |
|  |  |  |  |  | Cramps |  |
|  |  |  |  |  | Irrational behaviour |  |
|  |  |  |  |  | Coma |  |
|  |  |  |  |  | Seizures |  |
|  |  |  |  |  | Headache |  |
|  |  |  |  |  | Mean core temperature: 40 ± 0.9℃ |  |
| Bedno *et al*, 2014[28] | United States of America | 2005 - 2006 | Cross-sectional | US Army (8621 weight qualified and 834 Excess body fat Army trainees) | Prevalence rate: 0.7% |  |
|  |  |  |  |  | **Intrinsic risk factors** |  |
|  |  |  |  |  | **Age** |  |
|  |  |  |  |  | Age 20 – 24 years vs 18 – 19 years | OR: 0.96 (0.53 – 1.73) |
|  |  |  |  |  | Age ≥ 25 years vs 18 – 19 years | OR: 0.48 (0.14 – 1.59) |
|  |  |  |  |  | **Race** |  |
|  |  |  |  |  | Black vs white | OR:0.60 (0.18 – 1.94) |
|  |  |  |  |  | Other vs white | OR: 1.72 (0.89 – 3.35) |
|  |  |  |  |  | Tobacco smoking vs no smoking | OR: 0.83 (0.42 – 1.64) |
|  |  |  |  |  | Weight qualified vs Excess body fat | OR 3.98 (2.17 – 7.29) |
|  |  |  |  |  | Being unfit (failed step test) | OR: 2 (1.13 - 3.53) |
|  |  |  |  |  | **Extrinsic risk factors** |  |
|  |  |  |  |  | Combat roles vs combat support services | OR: 1.92 (1.08 – 3.44) |
| Armed Forces Health Surveillance Branch, 2015[19] | United States of America | 2014 | Cross-sectional (Descriptive) | US Armed Forces (344 cases of EHS and 1683 of other heat injury) | Incidence rate: 1.47/1000 person-years |  |
|  |  |  |  |  | **Potential intrinsic risk factors** |  |
|  |  |  |  |  | Younger age |  |
|  |  |  |  |  | Asian/Pacific Islanders |  |
|  |  |  |  |  | **Potential extrinsic risk factors** |  |
|  |  |  |  |  | Combat specific occupations |  |
|  |  |  |  |  | Army and Marine Corps members |  |
| Stacey *et al*, 2015[51] | United Kingdom | 2007 - 2014 | Retrospective cross-sectional | UK Army (361 reported cases of EHS) | **Intrinsic risk factors** |  |
|  |  |  |  |  | Un-acclimatised vs acclimatised | OR: 0.31 (0.15 – 0.66) |
|  |  |  |  |  | Age ≥ 30 years vs <30 years | OR: 0.60 (0.29 – 1.23) |
|  |  |  |  |  | Group paced vs self-paced | OR: 1.66 (0.86 – 3.17) |
|  |  |  |  |  | Lack of fitness vs physically fit | OR: 0.93 (0.30 – 2.38) |
|  |  |  |  |  | Dehydrated vs euhydrated /overhydrated | OR: 1.47 (0.76 – 2.82) |
|  |  |  |  |  | Previous EHI vs no EHI | OR: 0.72 (0.33 – 1.58) |
|  |  |  |  |  | Sleep deprived vs rest adequately | OR: 0.76 (0.37 – 1.56) |
|  |  |  |  |  | Intercurrent illness vs no illness | OR: 0.52 (0.26 – 1.05) |
|  |  |  |  |  | **Extrinsic risk factors** |  |
|  |  |  |  |  | Hot climate vs temperate climate (non-summer months) | OR: 1.38 (0.67 – 2.80) |
|  |  |  |  |  | Summer months vs temperate climate (non-summer months) | OR: 1.09 (0.62 – 1.91) |
|  |  |  |  |  | Recruits vs Senior rank and officers | OR 0.42 (0.18 - 0.99) |
|  |  |  |  |  | Occlusive clothing vs vented clothing | OR: 0.56 (0.34 – 0.93) |
| Armed Forces Health Surveillance Branch, 2016[20] | United States of America | 2015 | Cross-sectional (Descriptive) | US Armed Forces (417 cases of heat stroke and 1933 of other heat injury) | Incidence rate: 1.81/1000 person-years |  |
|  |  |  |  |  | **Potential intrinsic risk factors** |  |
|  |  |  |  |  | Younger age |  |
|  |  |  |  |  | Asian/Pacific Islanders |  |
|  |  |  |  |  | **Potential extrinsic risk factors** |  |
|  |  |  |  |  | Combat specific occupations |  |
|  |  |  |  |  | Army and Marine Corps members |  |
| Stacey *et al*, 2016[50] | United Kingdom | 2009 - 2013 | Cross-sectional | British Army; 565 cases of heat illness | Incidence rate: 0.76/1000 person-years* |  |
| Armed Forces Health Surveillance Branch, 2017[21] | United States of America | 2016 | Cross-sectional (Descriptive) | US Army (401 cases of heat stroke and 2135 of other heat injury) | Incidence rate: 1.96/1000 person-years |  |
|  |  |  |  |  | **Potential intrinsic risk factors** |  |
|  |  |  |  |  | Younger age |  |
|  |  |  |  |  | Asian/Pacific Islanders |  |
|  |  |  |  |  | **Potential extrinsic risk factors** |  |
|  |  |  |  |  | Combat specific occupations |  |
|  |  |  |  |  | Army and Marine Corps members |  |
|  |  |  |  |  | Recruit trainees |  |
| Deshwal *et al*, 2017[32] | India | 2012 – 2014 | Cross-sectional | 78 Indian special forces | **Potential intrinsic risk factors** |  |
|  |  |  |  |  | Male gender |  |
|  |  |  |  |  | Sleep deprivation |  |
|  |  |  |  |  | **Potential extrinsic risk factors** |  |
|  |  |  |  |  | Protective clothing |  |
|  |  |  |  |  | **Clinical features** |  |
|  |  |  |  |  | Confusion |  |
|  |  |  |  |  | Seizures |  |
|  |  |  |  |  | Coma |  |
|  |  |  |  |  | Violent behaviour |  |
|  |  |  |  |  | **Biochemical markers** |  |
|  |  |  |  |  | Hyponatremia |  |
|  |  |  |  |  | Hypokalaemia |  |
|  |  |  |  |  | Hypophosphatemia |  |
|  |  |  |  |  | Hypocalcaemia |  |
|  |  |  |  |  | Elevated urea |  |
|  |  |  |  |  | Elevated creatinine |  |
|  |  |  |  |  | Elevated LDH |  |
|  |  |  |  |  | Elevated aspartate aminotransferase |  |
|  |  |  |  |  | Elevated alanine aminotransferase |  |
| Nelson *et al*, 2017[43] | United States of America | 2011 - 2014 | Retrospective cohort study | Us Army | **Mild HI** |  |
|  |  |  |  | 48384 SCT tested soldiers of African American descent | **Intrinsic risk factors** |  |
|  |  |  |  |  | Female vs male | HR 1.76 (1.48 - 2.10) |
|  |  |  |  |  | Antipsychotics vs no antipsychotics | HR 3.25 (1.33 - 7.90) |
|  |  |  |  |  | Age ≥ 35 years vs ≤ 22years | HR: 1.32 (0.84 – 2.09) |
|  |  |  |  |  | Age 23 – 27 years vs ≤ 22years | HR: 1.22 (0.80 – 1.67) |
|  |  |  |  |  | Age 28 – 35 years vs ≤ 22years | HR: 0.91 (0.63 – 1.32) |
|  |  |  |  |  | BMI 25 -29.99 vs BMI < 25 | HR: 0.99 (0.82 – 1.20) |
|  |  |  |  |  | BMI ≥ 30 vs BMI < 25 | HR: 1.13 (0.73 – 1.22) |
|  |  |  |  |  | Army Physical Fitness Test score ≥ 270 vs < 270 | HR: 0.94 (0.73 – 1.22) |
|  |  |  |  |  | Tobacco use vs no tobacco use | HR: 1.16 (0.91 – 1.48) |
|  |  |  |  |  | Statins vs no statins | HR: 0.58 (0.08 – 4.18) |
|  |  |  |  |  | Antipsychotics vs no antipsychotics | HR: 3.25 (1.33 – 7.90) |
|  |  |  |  |  | Stimulants vs no stimulants | HR: 1.67 (0.41 – 6.73) |
|  |  |  |  |  | Positive SCT vs negative SCT | HR: 1.15 (0.84 – 1.56) |
|  |  |  |  |  | **HS** |  |
|  |  |  |  |  | Prior mild HI vs no prior mild HI | HR 17.7 (8.50 - 36.7) |
|  |  |  |  |  | BMI 25 -29.99 vs BMI < 25 | HR 2.91 (1.38 - 6.17) |
|  |  |  |  |  | BMI ≥ 30 vs BMI < 25 | HR 4.04 (1.72 - 9.45) |
|  |  |  |  |  | Female vs male | HR: 0.61 (0.31 – 1.19) |
|  |  |  |  |  | Age ≥ 35 years vs ≤ 22years | HR: 0.77 (0.25 – 2.43) |
|  |  |  |  |  | Age 23 – 27 years vs ≤ 22years | HR: 0.65 (0.22 – 1.89) |
|  |  |  |  |  | Age 28 – 35 years vs ≤ 22years | HR: 0.92 (0.39 – 2.14) |
|  |  |  |  |  | Army physical Fitness Test score ≥ 270 vs < 270 | HR: 1.05 (0.53 – 2.08) |
|  |  |  |  |  | Tobacco use vs no tobacco use | HR: 1.04 (0.54 – 1.99) |
|  |  |  |  |  | Statins vs no statins | HR: 2.23 (0.29 – 16.9) |
|  |  |  |  |  | Antipsychotics vs no antipsychotics | HR: 3.67 (0.48 – 27.8) |
|  |  |  |  |  | Stimulants vs no stimulants | HR: 5.19 (0.70 – 38.8 |
|  |  |  |  |  | Positive SCT vs negative SCT | HR: 1.11 (0.44 – 2.79) |
| Armed Forces Health Surveillance Branch, 2018[22] | United States of America | 2017 | Cross-sectional (Descriptive) | US Armed Forces (464 cases of heat stroke and 1699 of other heat injury) | Incidence rate: 1.79/1000 person-years |  |
|  |  |  |  |  | **Potential intrinsic risk factors** |  |
|  |  |  |  |  | Younger age |  |
|  |  |  |  |  | Asian/Pacific Islanders |  |
|  |  |  |  |  | **Potential extrinsic risk factors** |  |
|  |  |  |  |  | Combat specific occupations |  |
|  |  |  |  |  | Army and Marine Corps members |  |
| Nelson *et al*, 2018[44] | United States of America | 2011 - 2014 | Retrospective cohort study | 238,168 US Army soldiers | Prevalence rate: 1.4% |  |
|  |  |  |  |  | **Intrinsic risk factors** |  |
|  |  |  |  |  | Female | OR 2.14 (1.95 - 2.34) |
|  |  |  |  |  | Age ≤20 vs ≥ 25 years | OR 1.23 (1.08 - 1.40) |
|  |  |  |  |  | Age 21 – 22 vs 25 years | OR 1.16 (1.02 - 1.33) |
|  |  |  |  |  | Black vs white | OR: 0.94 (0.86 – 1.04) |
|  |  |  |  |  | Asian or Pacific Islander | OR: 0.93 (0.77 – 1.12) |
|  |  |  |  |  | Formally married vs married | OR 1.52 (1.08 - 2.14) |
|  |  |  |  |  | Army Physical fitness test score |  |
|  |  |  |  |  | < 215 vs 270 | OR: 1.10 (0.87 – 1.40) |
|  |  |  |  |  | 215 – 244 vs 270 | OR: 0.99 (0.79 – 1.25) |
|  |  |  |  |  | 245 – 269 vs 270 | OR: 0.84 (0.66 – 1.08) |
|  |  |  |  |  | Prior HI of other type vs none | OR 4.02 (2.67 - 6.03) |
|  |  |  |  |  | BMI < 18.5 vs 18.5 - 24.99 | OR 1.50 (1.01 - 2.21) |
|  |  |  |  |  | BMI 25 - 29.99 vs 18.5 - 24.99 | OR 1.10 (1.01 - 1.19) |
|  |  |  |  |  | BMI 30 vs BMI 18.5 - 24.5 | OR 1.41 (1.19 - 1.67) |
|  |  |  |  |  | Tobacco use vs none | OR 1.55 (1.37 -1.77) |
|  |  |  |  |  | NSAID use vs no NSAID use | OR 1.31 (1.05 - 1.64) |
|  |  |  |  |  | Alpha blocker use vs no alpha blocker use | OR 6.09 (0.84 – 44.1) |
|  |  |  |  |  | Opioid use vs no opioid use | OR 1.92 (1.08 - 3.41) |
|  |  |  |  |  | Amphetamine use vs no amphetamine use | OR 0.70 (0.18 – 2.86) |
|  |  |  |  |  | Methylphenidate use vs no methylphenidate use | OR 5.68 (1.41 - 22.9) |
|  |  |  |  |  | **Extrinsic risk factors** |  |
|  |  |  |  |  | Higher pay grade vs lower enlisted | OR: 1.44 (0.76 – 2.70) |
|  |  |  |  |  | Armed Forces Qualifying Test score |  |
|  |  |  |  |  | 42 vs 75 | OR 1.26 (1.12 - 1.42) |
|  |  |  |  |  | 43 – 56 vs 75 | OR 1.14 (1.01 - 1.27) |
|  |  |  |  |  | First four months of service vs latter service | OR 2.97 (2.60 - 3.39) |
|  |  |  |  |  | Spring vs winter | OR 5.90 (4.56 - 7.63) |
|  |  |  |  |  | Summer vs winter | OR 22.1 (17.3 - 28.2) |
|  |  |  |  |  | Fall vs winter | OR 4.95 (3.82 - 6.42) |
|  |  |  |  |  | **SHI** |  |
|  |  |  |  |  | **Intrinsic risk factors** |  |
|  |  |  |  |  | Female | OR 1.66 (1.40 - 1.98) |
|  |  |  |  |  | Black race vs white | OR 1.72 (1.46 - 2.03) |
|  |  |  |  |  | Never married vs married | OR 1.29 (1.05 - 1.59) |
|  |  |  |  |  | Prior HI of other type vs none | OR 1.77 (1.00 - 3.13) |
|  |  |  |  |  | BMI < 18.5 vs 18.5 - 24.99 | OR 2.26 (1.16 - 4.39) |
|  |  |  |  |  | BMI 25 - 29.99 vs 18.5 - 24.99 | OR 1.29 (1.10 - 1.51) |
|  |  |  |  |  | BMI 30 vs BMI 18.5 - 24.5 | OR 1.94 (1.47 - 2.56) |
|  |  |  |  |  | Army Physical fitness test score |  |
|  |  |  |  |  | < 215 vs 245 - 269 | OR: 1.34 (0.84 – 2.12) |
|  |  |  |  |  | 215 – 244 vs 245 - 269 | OR: 1.17 (0.75 – 1.81) |
|  |  |  |  |  | 270 vs 245 - 269 | OR: 1.32 (0.83 – 2.10) |
|  |  |  |  |  | Tobacco use vs no tobacco use | OR: 1.19 (0.95 – 1.49) |
|  |  |  |  |  | NSAID use vs no NSAID use | OR: 0.84 (0.49 – 1.44) |
|  |  |  |  |  | Amphetamine use vs no amphetamine use | OR: 2.93 (0.73 – 11.8) |
|  |  |  |  |  | **Extrinsic risk factors** |  |
|  |  |  |  |  | First four months of service vs latter service | OR 1.79 (1.41 - 2.26) |
|  |  |  |  |  | Lower enlisted vs higher pay grade | OR: 2.76 (0.61 – 12.4) |
|  |  |  |  |  | Armed Forces Qualifying Test score |  |
|  |  |  |  |  | 42 vs 75 | OR: 1.04 (0.84 – 1.30) |
|  |  |  |  |  | 43 – 56 vs 75 | OR: 0.92 (0.7 – 1.14) |
|  |  |  |  |  | 57 – 74 vs 75 | OR: 0.93 (0.75 – 1.15) |
|  |  |  |  |  | Spring vs winter | OR 5.55 (3.60 - 8.55) |
|  |  |  |  |  | Summer vs winter | OR 16.3 (10.8 - 24.6) |
|  |  |  |  |  | Fall vs winter | OR 4.83 (3.12 - 7.45) |
| Nutong *et al* 2018[45] | Thailand | 2013 (3 months) | Cohort study | 809 newly inducted Royal Thai conscripts | Prevalence rate: 6.6 |  |
|  |  |  |  |  | **Intrinsic risk factors** |  |
|  |  |  |  |  | BMI ≥30 vs BMI 18.5 - 24.5 | IRR 2.66 (1.01 -7.03) |
|  |  |  |  |  | Current smoker vs never smoked | IRR: 1.58 (0.73 – 3.43) |
|  |  |  |  |  | Ex-smoker vs never smoked | IRR: 2.14 (0.70 – 6.55) |
| Singer *et al*,2018[47] | United States of America | 1992 - 2012 | Retrospective cohort study | US Army | **Intrinsic risk factors** |  |
|  |  |  |  | 15081 SCT positive personnel and 60320 SCT negative personnel | SCT positive vs SCT negative | HR: 1.24 (1.06 - 1.45) |
|  |  |  |  |  | Age: ≥30 years vs 18 - 19 years | HR: 1.5 (1.07 - 2.33) |
|  |  |  |  |  | Female vs male | HR: 1.36 (1.17 - 1.59) |
|  |  |  |  |  | **Extrinsic risk factors** |  |
|  |  |  |  |  | Marines vs Army | HR: 1.51 (1.22 - 1.88) |
|  |  |  |  |  | **Occupation** |  |
|  |  |  |  |  | Combat vs Repair/engineer | HR: 1.57 (1.15 - 2.13) |
|  |  |  |  |  | Healthcare vs Repair/engineer | HR: 1.42 (1.08 - 1.87) |
|  |  |  |  |  | Other vs Repair/engineer | HR: 1.62 (1.31 - 2.00) |
| Armed Forces Health Surveillance Branch, 2019[23] | United States of America | 2018 | Cross-sectional (Descriptive) | US Armed Forces (578 cases of EHS and 2214 cases of HE) | Incidence rate: 2.15/1000 person-years |  |
|  |  |  |  |  | **Potential intrinsic risk factors** |  |
|  |  |  |  |  | Younger age |  |
|  |  |  |  |  | Asian/Pacific Islanders |  |
|  |  |  |  |  | **Potential extrinsic risk factors** |  |
|  |  |  |  |  | Combat specific occupations |  |
|  |  |  |  |  | Army and Marine Corps members |  |
| Barnes *et al*, 2019[26] | United States of America | 2014 - 2018 | Retrospective cohort study | US Army | Incidence rate: 3.6/10,000 BCT person-weeks |  |
|  |  |  |  | 352,739 recruits in basic combat training | Prevalence rate: 0.3% |  |
|  |  |  |  |  | **Intrinsic risk factors** |  |
|  |  |  |  |  | Female | RR: 2.3 (2.1 – 2.6) |
|  |  |  |  |  | Non- Hispanic black vs non-Hispanic white | RR:1.4 (1.2 – 1.6) |
|  |  |  |  |  | **Extrinsic risk factors** |  |
|  |  |  |  |  | National Guard soldiers vs soldiers in active duty | RR: 1.1 (1.0 -1.2) |
| King *et al*, 2019[40] | United States of America | 2012 - 2015 | Cross-sectional | 179 Marine Corps | **Potential intrinsic risk factor** |  |
|  |  |  |  |  | Prior illness |  |
|  |  |  |  |  | **Clinical features** |  |
|  |  |  |  |  | Mean core temperature: 40.4℃ |  |
|  |  |  |  |  | **Biochemical markers** |  |
|  |  |  |  |  | Hyperglycaemia |  |
| Donham *et al,* 2020 [34] | United Sates of America | 2007 - 2014 | Cross-sectional | 48 US military service members | **Biochemical markers** |  |
|  |  |  |  |  | Elevated alanine aminotransferase |  |
|  |  |  |  |  | Elevated aspartate aminotransferase |  |
|  |  |  |  |  | Elevated creatine phosphokinase |  |
| Gardner *et al,* 2020 [36] | United Kingdom | NS | Cross-sectional | 59 UK military members with a history of EHI | **Potential intrinsic risk factors** |  |
|  |  |  |  |  | Alcohol consumption |  |
|  |  |  |  |  | Intercurrent illness |  |
|  |  |  |  |  | dehydration |  |
|  |  |  |  |  | **Potential extrinsic risk factors** |  |
|  |  |  |  |  | Extreme heat |  |
| Ward *et al*,2020[54] | United Sates of America | 2008– 2014 | Cross-sectional | 2216 US military service members | **Biochemical markers** |  |
|  |  |  |  |  | Elevated alanine aminotransferase |  |
|  |  |  |  |  | Elevated aspartate aminotransferase |  |
|  |  |  |  |  | Elevated creatinine |  |
|  |  |  |  |  | Elevated chloride |  |
|  |  |  |  |  | Elevated calcium |  |

* Incidence rates were converted to per 1000 person-years; ‡Two axillary temperature reading (37.6°C and 39°C) were excluded from the sample; NS: Not stated
