# Supplementary material for: Epidemiology of Exertional Heat Illness in the Military: A Systematic Review of Observational Studies
Source: Int J Environ Res Public Health. 2020 Sep 25;17(19):7037. doi: 10.3390/ijerph17197037 (PMC7579124; doi:10.3390/ijerph17197037)
Supplement: Supplementary file 1 [file ijerph-17-07037-s001.zip › ijerph-927752-Supplementary/Supplementary Table 3.docx]

**Table S3: Characteristics of the risk factors associated with EHI supported by the effect sizes (strength of association)**

| **Classification** |  | **Risk factors** | **OR/RR/HR/IDR (95% CI)** | **Type of HI** | **Year of study** | **Location** | **Author and year** |
| --- | --- | --- | --- | --- | --- | --- | --- |
| **Intrinsic** | **Sociodemographic factors** | **Age** |  |  |  |  |  |
|  |  | Older age (≥ 20 years vs < 20 years) | OR: 0.91 (0.52 – 1.58) | EHI | 2005 - 2006 | USA | Bedno *et al.*, 2010[33] |
|  |  | Older age (20 – 24 years vs 18 – 19 years) | OR: 0.96 (0.53 – 1.73) | EHI | 2005 - 2006 | USA | Bedno *et al.*, 2014[34] |
|  |  | Older age (≥ 25 years vs 18 – 19 years) | OR: 0.48 (0.14 – 1.59) |  |  |  |  |
|  |  | Older age (≥ 30 years vs <30 years) | OR: 0.60 (0.29 – 1.23) | EHI | 2007 - 2014 | UK | Stacey *et al.*, 2015[57] |
|  |  | Older age (≥30 years vs 18 - 19 years) | HR: 1.5 (1.07 - 2.33)* | EHI | 1992 - 2012 | USA | Singer *et al.*, 2018[53] |
|  |  | Older age (≥ 35 years vs ≤ 22years) | HR: 1.32 (0.84 – 2.09) | MHI | 2011 - 2014 | USA | Nelson *et al.*, 2017[49] |
|  |  | Older age (28 – 35 years vs ≤ 22years) | HR: 0.91 (0.63 – 1.32) |  |  |  |  |
|  |  | Older age (23 – 27 years vs ≤ 22years) | HR: 1.22 (0.80 – 1.67) |  |  |  |  |
|  |  | Older age (≥ 35 years vs ≤ 22years) | HR: 0.77 (0.25 – 2.43) | HS |  |  |  |
|  |  | Older age (28 – 35 years vs ≤ 22years) | HR: 0.92 (0.39 – 2.14) |  |  |  |  |
|  |  | Older age (23 – 27 years vs ≤ 22years) | HR: 0.65 (0.22 – 1.89) |  |  |  |  |
|  |  | Younger age (≤20 vs 25 years) | OR 1.23 (1.08 - 1.40)* | MHI | 2011 - 2014 | USA | Nelson *et al.*, 2018[50] |
|  |  | Younger age (21 – 22 vs 25 years) | OR 1.16 (1.02 - 1.33)* |  |  |  |  |
|  |  | Younger age (23 – 24 vs 25 years) | OR 1.05 (0.90 – 1.22) |  |  |  |  |
|  |  | Younger age (≤20 vs 25 years) | OR 0.89 (0.71 – 1.12) | SHI |  |  |  |
|  |  | Younger age (21 – 22 vs 25 years) | OR 0.93 (0.73 – 1.17) |  |  |  |  |
|  |  | Younger age (23 – 24 vs 25 years) | OR 0.92 (0.71 – 1.18) |  |  |  |  |
|  |  | **Gender** |  |  |  |  |  |
|  |  | Female vs male | IDR: 1.18 (1.09 - 1.27)* | EHI | 1980 - 2002 | USA | Carter *et al.,* 2005[13] |
|  |  | Female vs male | HR 1.76 (1.48 - 2.10)* | MHI | 2011 - 2014 | USA | Nelson *et al.*, 2017[49] |
|  |  | Female vs male | HR: 0.61 (0.31 – 1.19) | HS |  |  |  |
|  |  | Female vs male | OR 2.14 (1.95 - 2.34)* | MHI | 2011 - 2014 | USA | Nelson *et al.*, 2018[50] |
|  |  | Female vs male | OR 1.66 (1.40 - 1.98)* | SHI |  |  |  |
|  |  | Female vs male | HR: 1.36 (1.17 - 1.59)* | EHI | 1992 - 2012 | USA | Singer *et al.*, 2018[53] |
|  |  | Female vs male | RR: 2.3 (2.1 – 2.6)* | EHI | 2014 - 2018 | USA | Barnes *et al.*, 2019[32] |
|  |  | **Marital status** |  |  |  |  |  |
|  |  | Never married | OR 1.11 (0.99 – 1.25) | MH1 | 2011 - 2014 | USA | Nelson *et al.*, 2018[50] |
|  |  | Formerly married | OR 1.52 (1.08 – 2.14)* |  |  |  |  |
|  |  | Never married | OR 1.29 (1.05 – 1.59)* | SHI |  |  |  |
|  |  | Formerly married | OR 0.58 (0.22 – 1.58) |  |  |  |  |
|  |  | **Race** |  |  |  |  |  |
|  |  | Non-whites vs whites | OR: 1.7 (NS)* | EHI | 1988 - 1992 | USA | Gardner *et al.,* 1996[41] |
|  |  | African and Hispanic vs Caucasians | IDR: 0.76 (0.71 - 0.82)* | EHI | 1980 - 2002 | USA | Carter *et al.*, 2005[13] |
|  |  | Black vs white and other | OR: 0.75 (0.27 – 2.10) | EHI | 2005 – 2006 | USA | Bedno *et al.*, 2010[33] |
|  |  | Black vs white | OR:0.60 (0.18 – 1.94) | EHI | 2005 – 2006 | USA | Bedno *et al.*, 2014[34] |
|  |  | Other vs white | OR: 1.72 (0.89 – 3.35) |  |  |  |  |
|  |  | Black vs white | OR: 0.94 (0.86 – 1.04) | MHI | 2011 - 2014 | USA | Nelson *et al.*, 2018[50] |
|  |  | Black vs white | OR 1.72 (1.46 - 2.03)* | SHI |  |  |  |
|  |  | Non- Hispanic black vs non-Hispanic white | RR:1.4 (1.2 – 1.6)* | EHI | 2014 - 2018 | USA | Barnes *et al.*, 2019[32] |
|  | **Physiological and behavioural factors** |  |  |  |  |  |  |
|  |  | **Acclimatization** |  |  |  |  |  |
|  |  | Un-acclimatised vs acclimatised | OR: 0.31 (0.15 – 0.66) | EHI | 2007 - 2014 | UK | Stacey *et al.*, 2015[57] |
|  |  | **Motivation** |  |  |  |  |  |
|  |  | Group paced vs self-paced | OR: 1.66 (0.86 – 3.17) | EHI | 2007 - 2014 | UK | Stacey *et al.*, 2015[57] |
|  |  | Exercise intensity **(**Running the extra kilometre) | RR 3.4 (1.3 - 9.0)* | EHI | 1982 | Ecuador | Harris *et al.*, 1985[44] |
|  |  | Sleep deprived vs rest adequately | OR: 0.76 (0.37 – 1.56) | EHI | 2007 - 2014 | UK | Stacey *et al.,* 2015[57] |
|  |  | **Hydration status** |  |  |  |  |  |
|  |  | Dehydrated vs euhydrated /overhydrated | OR: 1.47 (0.76 – 2.82) | EHI | 2007 - 2014 | UK | Stacey *et al.,* 2015[57] |
|  | **Anthropometric factors** |  |  |  |  |  |  |
|  |  | **Overweight/obesity** |  |  |  |  |  |
|  |  | Obesity (BMI < 27 Kgm^-2^ vs BMI > 27 Kgm^-2^) | OR 4.3 (NS)* | EHI | 1992 - 1994 | Singapore | Chung and Pin 1996[37] |
|  |  | Overweight (BMI 22 - <26 Kgm^-2^ VS <22 Kgm^-2^) | OR: 1.9 (NS)* | EHI | 1988 - 1992 | USA | Gardener *et al.,* 1996[41] |
|  |  | Obesity (BMI >=26 Kgm^-2^ VS <22 Kgm^-2^) | OR: 1.6 (NS)* |  |  |  |  |
|  |  | *Men* |  | EHI | 1986 - 1996 | USA | Wallace *et al.*, 2006[59] |
|  |  | Overweight (BMI 22 - <26 Kgm^-2^ VS <22 Kgm^-2^) | OR 1.51 (1.17 - 1.93) * |  |  |  |  |
|  |  | Obesity (BMI >=26 Kgm^-2^ VS <22 Kgm^-2^) | OR 2.10 (1.59 - 2.78)* |  |  |  |  |
|  |  | Excess body fat vs no excess body fat | OR: 3.63 (1.92 - 6.85)* | EHI | 2005 - 2006 | USA | Bedno *et al.,* 2010[33] |
|  |  | Weight qualified vs Excess body fat | OR 3.98 (2.17 – 7.29)* | EHI | 2005 – 2006 | USA | Bedno *et al.*, 2014[34] |
|  |  | Overweight (BMI 25 -29.99 vs BMI < 25 Kgm^-2^) | HR: 0.99 (0.82 – 1.20) | MHI | 2011 - 2014 | USA | Nelson *et al.*, 2017[49] |
|  |  | Obesity (BMI ≥ 30 vs BMI < 25 Kgm^-2^) | HR: 1.13 (0.73 – 1.22) |  |  |  |  |
|  |  | Overweight (BMI 25 -29.99 vs BMI < 25 Kgm^-2^) | HR 2.91 (1.38 - 6.17)* | HS |  |  |  |
|  |  | Obesity (BMI ≥ 30 vs BMI < 25 Kgm^-2^) | HR 4.04 (1.72 - 9.45)* |  |  |  |  |
|  |  | Underweight (BMI < 18.5 vs 18.5 - 24.99 Kgm^-2^) | OR 1.50 (1.01 - 2.21)* | MHI | 2011 - 2014 | USA | Nelson *et al.*, 2018[50] |
|  |  | Overweight (BMI 25 - 29.99 vs 18.5 - 24.99 Kgm^-2^) | OR 1.10 (1.01 - 1.19)* |  |  |  |  |
|  |  | Obesity (BMI 30 vs BMI 18.5 - 24.5 Kgm^-2^) | OR 1.41 (1.19 - 1.67)* |  |  |  |  |
|  |  | Underweight (BMI < 18.5 vs 18.5 - 24.99 Kgm^-2^) | OR 2.26 (1.16 - 4.39)* | SHI |  |  |  |
|  |  | Overweight (BMI 25 - 29.99 vs 18.5 - 24.99 Kgm^-2^) | OR 1.29 (1.10 - 1.51)* |  |  |  |  |
|  |  | Obesity (BMI 30 vs BMI 18.5 - 24.5 Kgm^-2^) | OR 1.94 (1.47 - 2.56)* |  |  |  |  |
|  |  | Obesity (BMI ≥30 vs BMI 18.5 - 24.5) | IRR 2.66 (1.01 -7.03)* | EHI | 2013 | Thailand | Nutong *et al.,* 2018[51] |
|  | **Fitness factors** |  |  |  |  |  |  |
|  |  | **Physical fitness** |  |  |  |  |  |
|  |  | *1.5-mile physical fitness test* |  | EHI | 1988 - 1992 | USA | Gardener *et al.*, 1996[41] |
|  |  | Run time of 10 mins - < 12 mins vs < 10 mins | OR: 1.1 (NS)* |  |  |  |  |
|  |  | Run time of >= 12 mins vs < 10mins | OR: 3.4 (NS)* |  |  |  |  |
|  |  | *3-mile physical fitness test* |  |  |  |  |  |
|  |  | Run time of 20 mins - < 23 mins vs < 20 mins | OR: 2.1 (NS)* |  |  |  |  |
|  |  | Run time of >= 23 mins vs < 20 mins | OR: 4.2 (NS)* |  |  |  |  |
|  |  | *Women* |  | EHI | 1986 - 1996 | USA | Wallace *et al.*, 2006[59] |
|  |  | Run time of >= 6.9 mins vs < 5.8 mins | OR 5.30 (1.59 - 17.64)* |  |  |  |  |
|  |  | *Men* |  |  |  |  |  |
|  |  | Run time of 10.3 - < 11.7 mins vs < 10.3 mins | OR 1.51 (1.10 - 2.06)* |  |  |  |  |
|  |  | Run time of 11.7 - < 12.9 vs <10.3mins | OR 3.62 (2.61 - 5.03)* |  |  |  |  |
|  |  | Run time of >= 12.9 mins vs < 10.3 mins | OR 5.61 (3.73 - 8.45)* |  |  |  |  |
|  |  | Being unfit (failed step test) | OR: 2 (1.13 - 3.53)* | EHI | 2005 – 2006 | USA | Bedno *et al.*, 2014[34] |
|  |  | Lack of fitness vs physically fit | OR: 0.93 (0.30 – 2.38) | EHI | 2002 - 2014 | UK | Stacey *et al.*, 2015[57] |
|  |  | Army Physical Fitness Test score ≥ 270 vs < 270 | HR: 0.94 (0.73 – 1.22) | MHI | 2011 - 2014 | USA | Nelson *et al.*, 2017[49] |
|  |  | Army physical Fitness Test score ≥ 270 vs < 270 | HR: 1.05 (0.53 – 2.08) | HS |  |  |  |
|  |  | Army Physical fitness test score |  | MHI | 2011 - 2014 | USA | Nelson *et al.*, 2018[50] |
|  |  | < 215 vs 270 | OR: 1.10 (0.87 – 1.40) |  |  |  |  |
|  |  | 215 – 244 vs 270 | OR: 0.99 (0.79 – 1.25) |  |  |  |  |
|  |  | 245 – 269 vs 270 | OR: 0.84 (0.66 – 1.08) |  |  |  |  |
|  |  | No fitness data test score | OR 1.21 (0.99 – 1.48) |  |  |  |  |
|  |  | Army Physical fitness test score |  | SHI |  |  |  |
|  |  | < 215 vs 245 - 269 | OR: 1.34 (0.84 – 2.12) |  |  |  |  |
|  |  | 215 – 244 vs 245 - 269 | OR: 1.17 (0.75 – 1.81) |  |  |  |  |
|  |  | 270 vs 245 - 269 | OR: 1.32 (0.83 – 2.10) |  |  |  |  |
|  |  | No fitness data test score | OR: 2.2 (1.52 – 3.17)* |  |  |  |  |
|  | **Medical history** |  |  |  |  |  |  |
|  |  | **Previous heat injury** |  |  |  |  |  |
|  |  | Previous EHI vs no EHI | OR: 0.72 (0.33 – 1.58) | EHI | 2007 - 2014 | UK | Stacey *et al.*, 2015[57] |
|  |  | Prior mild HI vs no prior mild HI | HR 17.7 (8.50 - 36.7)* | HS | 2011 - 2014 | USA | Nelson *et al.*, 2017[49] |
|  |  | Prior HI of other type vs none | OR 1.77 (1.00 - 3.13)* | SHI | 2011 - 2014 | USA | Nelson *et al.*, 2018[50] |
|  |  | **Pre-existing illness** |  |  |  |  |  |
|  |  | Intercurrent illness vs no illness | OR: 0.52 (0.26 – 1.05) | EHI | 2007 - 2014 | UK | Stacey *et al.,* 2015[57] |
|  |  | **Genetics** |  |  |  |  |  |
|  |  | Positive SCT vs negative SCT | HR: 1.15 (0.84 – 1.56) | MHI | 2011 - 2014 | USA | Nelson *et al.*, 2017[49] |
|  |  | Positive SCT vs negative SCT | HR: 1.11 (0.44 – 2.79) | HS |  |  |  |
|  |  | Positive SCT vs SCT negative | HR: 1.24 (1.06 - 1.45)* | EHI | 1992 - 2012 | USA | Singer *et al.*, 2018 [53] |
|  | **Medications and lifestyle** |  |  |  |  |  |  |
|  |  | **Medications** |  |  |  |  |  |
|  |  | Antipsychotics vs no antipsychotics | HR 3.25 (1.33 - 7.90)* | MHI | 2011 - 2014 | USA | Nelson *et al.*, 2017[49] |
|  |  | Stimulants vs no stimulants | HR: 1.67 (0.41 – 6.73) |  |  |  |  |
|  |  | Statins vs no statins | HR: 0.58 (0.08 – 4.18) |  |  |  |  |
|  |  | Antipsychotics vs no antipsychotics | HR: 3.67 (0.48 – 27.8) | HS |  |  |  |
|  |  | Stimulants vs no stimulants | HR: 5.19 (0.70 – 38.8 |  |  |  |  |
|  |  | Statins vs no statins | HR: 2.23 (0.29 – 16.9) |  |  |  |  |
|  |  | NSAID use vs no NSAID use | OR 1.31 (1.05 - 1.64)* | MHI | 2011 - 2014 | USA | Nelson *et al.*, 2018[50] |
|  |  | Alpha blocker use vs no alpha blocker use | OR 6.09 (0.84 – 44.1) |  |  |  |  |
|  |  | Opioid use vs none | OR 1.92 (1.08 - 3.41)* |  |  |  |  |
|  |  | Amphetamine use vs no amphetamine use | OR 0.70 (0.18 – 2.86) |  |  |  |  |
|  |  | Methylphenidate use vs no methylphenidate use | OR 5.68 (1.41 - 22.9)* |  |  |  |  |
|  |  | NSAID use vs no NSAID use | OR: 0.84 (0.49 – 1.44) | SHI |  |  |  |
|  |  | Amphetamine use vs no amphetamine use | OR: 2.93 (0.73 – 11.8) |  |  |  |  |
|  |  | **Alcohol and tobacco use** |  |  |  |  |  |
|  |  | Tobacco smoking vs no smoking | OR: 0.83 (0.42 – 1.64) | EHI | 2005 – 2006 | USA | Bedno *et al.*, 2014[34] |
|  |  | Tobacco use vs no tobacco use | HR: 1.16 (0.91 – 1.48) | MHI | 2011 - 2014 | USA | Nelson *et al.*, 2017[49] |
|  |  | Tobacco use vs no tobacco use | HR: 1.04 (0.54 – 1.99) | HS |  |  |  |
|  |  | Tobacco use vs no tobacco use | OR 1.55 (1.37 -1.77)* | MHI | 2011 - 2014 | USA | Nelson *et al.*, 2018[50] |
|  |  | Tobacco use vs no tobacco use | OR: 1.19 (0.95 – 1.49) | SHI |  |  |  |
|  |  | Current smoker vs never smoked | IRR: 1.58 (0.73 – 3.43) | EHI | 2013 | Thailand | Nutong *et al.,* 2018[51] |
|  |  | Ex-smoker vs never smoked | IRR: 2.14 (0.70 – 6.55) |  |  |  |  |
| **Extrinsic** | **Training factors** |  |  |  |  |  |  |
|  |  | **Clothing and equipment** |  |  |  |  |  |
|  |  | Occlusive clothing vs vented clothing | OR: 0.56 (0.34 – 0.93)* | EHI | 2007 - 2014 | UK | Stacey *et al.,* 2015[57] |
|  |  | **Service units and roles** |  |  |  |  |  |
|  |  | First year cadets vs other cadets | RR 3.0 (1.3 - 7.0)* | EHI | 1982 | Ecuador | Harris *et al.*, 1985[44] |
|  |  | Infantry vs gun crew | IDR: 2.67 (1.71 - 2.89)* | EHI | 1980 - 2002 | USA | Carter *et al.,* 2005[13] |
|  |  | Combat roles vs combat support services | OR: 1.92 (1.08 – 3.44)* | EHI | 2005 – 2006 | USA | Bedno *et al.*, 2014[34] |
|  |  | Recruits vs Senior rank and officers | OR 0.42 (0.18 - 0.99) | EHI | 2007 - 2014 | UK | Stacey *et al.,* 2015[57] |
|  |  | Combat vs Repair/engineer | HR: 1.57 (1.15 - 2.13)* | EHI | 1992 - 2012 | USA | Singer *et al.*, 2018 [53] |
|  |  | Healthcare vs Repair/engineer | HR: 1.42 (1.08 - 1.87)* |  |  |  |  |
|  |  | Other vs Repair/engineer | HR: 1.62 (1.31 - 2.00)* |  |  |  |  |
|  |  | Marines vs Army | HR: 1.51 (1.22 - 1.88)* |  |  |  |  |
|  |  | National Guard soldiers vs Soldiers in active duty | RR: 1.1 (1.0 -1.2)* | EHI | 2014 - 2018 | USA | Barnes *et al.*, 2019[32] |
|  | **Environmental factors** |  |  |  |  |  |  |
|  |  | **Hot environmental conditions** |  |  |  |  |  |
|  |  | Increasing WGBT | OR: 1.11 °F¯¹ (1.10 - 1.13)* | EHI | 1979 - 1997 | USA | Wallace *et al.*, 2005[58] |
|  |  | WBGT at time of event | OR: 1.10 °F¯¹ (1.08 - 1.11)* |  |  |  |  |
|  |  | Average WBGT on the previous day | OR: 1.03 °F¯¹ (1.02 - 1.05)* |  |  |  |  |
|  |  | Hot climate vs temperate climate (non-summer months) | OR: 1.38 (0.67 – 2.80) | EHI | 2007 - 2014 | UK | Stacey *et al.,* 2015[57] |
|  |  | Summer months vs temperate climate (non-summer months) | OR: 1.09 (0.62 – 1.91) | MHI | 2011 - 2014 | USA | Nelson *et al.*, 2018[50] |
|  |  | Spring vs winter | OR 5.90 (4.56 - 7.63)* |  |  |  |  |
|  |  | Summer vs winter | OR 22.1 (17.3 - 28.2)* |  |  |  |  |
|  |  | Fall vs winter | OR 4.95 (3.82 - 6.42)* |  |  |  |  |
|  |  | Spring vs winter | OR 5.55 (3.60 - 8.55)* | SHI |  |  |  |
|  |  | Summer vs winter | OR 16.3 (10.8 - 24.6)* |  |  |  |  |
|  |  | Fall vs winter | OR 4.83 (3.12 - 7.45)* |  |  |  |  |

* Significant results; RR: Relative risk; OR: Odds ratio, IDR: Incidence density ratio; HR: Hazard ratio; SCT: Sickle cell trait; BMI: Body mass index; IRR: Incidence risk ratio; NS: Not stated; SHI: Severe heat illness; MHI: Mild heat illness; HS: Heat stroke; USA: United States of America; UK: United Kingdom
