# Supplementary material for: Epidemiology of Exertional Heat Illness in the Military: A Systematic Review of Observational Studies
Source: Int J Environ Res Public Health. 2020 Sep 25;17(19):7037. doi: 10.3390/ijerph17197037 (PMC7579124; doi:10.3390/ijerph17197037)
Supplement: Supplementary file 1 [file ijerph-17-07037-s001.zip › ijerph-927752-Supplementary/Supplementary Table 4.docx]

**Table S4: Quality assessment of included studies using the modified quality assessment tool for studies with diverse designs (QATSDD)**

| **QATSDD Criteria** | **1** | **2** | **3** | **4** | **5** | **6** | **7** | **8** | **9** | **10** | **11** | **12** | **Total score** | **% of total score** |
| --- | --- | --- | --- | --- | --- | --- | --- | --- | --- | --- | --- | --- | --- | --- |
| Kerstein *et al’*, 1984[39] | 1 | 3 | 2 | 2 | 1 | 3 | 0 | 0 | 3 | 3 | 0 | 0 | 18/36 | 50 |
| Harris *et al,* 1985 [38] | 0 | 2 | 1 | 0 | 2 | 3 | 0 | 0 | 3 | 3 | 0 | 0 | 14/36 | 38.9 |
| Bricknell, 1994[29] | 3 | 3 | 3 | 2 | 2 | 3 | 3 | 0 | 3 | 3 | 0 | 0 | 25/36 | 69.4 |
| Dickinson, 1994[33] | 0 | 1 | 2 | 0 | 1 | 3 | 0 | 0 | 3 | 2 | 0 | 2 | 14/36 | 38.9 |
| Shieh *et al,* 1995[46] | 2 | 2 | 3 | 2 | 0 | 3 | 2 | 3 | 3 | 3 | 3 |  | 26/36 | 72.2 |
| Bricknell, 1996[30] | 3 | 3 | 3 | 2 | 2 | 3 | 3 | 0 | 3 | 2 | 0 | 0 | 24/36 | 66.7 |
| Chung and Pin, 1996[31] | 3 | 3 | 2 | 3 | 3 | 3 | 3 | 0 | 3 | 3 | 2 | 0 | 28/36 | 77.8 |
| Gardner *et al,* 1996[35] | 3 | 3 | 2 | 3 | 2 | 3 | 3 | 3 | 3 | 3 | 2 | 3 | 33/36 | 91.7 |
| Lin *et al,* 2003[41] | 3 | 3 | 2 | 3 | 2 | 3 | 3 | 3 | 3 | 3 | 2 | 3 | 33/36 | 91.7 |
| Smalley *et al,* 2003[49] | 3 | 3 | 2 | 2 | 2 | 3 | 0 | 2 | 3 | 3 | 2 | 0 | 25/36 | 69.4 |
| Hakre *et al,* 2004[37] | 3 | 3 | 3 | 2 | 2 | 3 | 1 | 2 | 3 | 3 | 2 | 3 | 30/36 | 83.3 |
| Sonna *et al,* 2004[55] | 3 | 3 | 2 | 2 | 0 | 3 | 0 | 2 | 3 | 3 | 2 | 3 | 26/36 | 72.2 |
| Carter *et al,* 2005[13] | 3 | 3 | 3 | 2 | 2 | 3 | 1 | 2 | 3 | 3 | 2 | 0 | 27/36 | 75 |
| Wallace *et al,* 2005[52] | 3 | 3 | 3 | 2 | 2 | 3 | 2 | 3 | 3 | 3 | 2 | 2 | 31/36 | 86.1 |
| Wallace *et al*, 2006[53] | 3 | 3 | 3 | 2 | 2 | 3 | 1 | 2 | 3 | 3 | 2 | 2 | 29/36 | 80.6 |
| Sithinamsuwan *et al,* 2009[48] | 3 | 3 | 3 | 2 | 2 | 3 | 1 | 3 | 3 | 3 | 2 | 0 | 28/36 | 77.8 |
| Bedno *et al,* 2010 [27] | 3 | 3 | 3 | 2 | 2 | 3 | 3 | 3 | 3 | 3 | 3 | 3 | 34/36 | 94.4 |
| AFHSB, 2011[24] | 2 | 1 | 2 | 0 | 0 | 1 | 0 | 0 | 2 | 3 | 0 | 2 | 13/36 | 36.1 |
| AFHSB, 2012[17] | 2 | 1 | 2 | 0 | 0 | 1 | 0 | 0 | 2 | 3 | 0 | 2 | 13/36 | 36.1 |
| AFHSB, 2013[18] | 2 | 2 | 2 | 0 | 0 | 2 | 0 | 0 | 2 | 3 | 0 | 2 | 15/36 | 41.7 |
| AFHSB, 2014[25] | 2 | 2 | 2 | 0 | 0 | 2 | 0 | 0 | 2 | 3 | 0 | 2 | 15/36 | 41.7 |
| Bedno *et al,* 2014[28] | 3 | 3 | 3 | 2 | 2 | 3 | 3 | 3 | 3 | 3 | 3 | 3 | 34/36 | 94.4 |
| Abriat *et al,* 2015[16] | 3 | 3 | 3 | 2 | 2 | 3 | 1 | 2 | 3 | 3 | 2 | 0 | 27/36 | 75 |
| AFHSB, 2015[19] | 3 | 2 | 2 | 0 | 0 | 2 | 0 | 0 | 2 | 3 | 0 | 2 | 16/36 | 44.4 |
| Stacey *et al,*2015[51] | 3 | 3 | 3 | 3 | 3 | 3 | 2 | 3 | 3 | 3 | 3 | 3 | 35/36 | 97.2 |
| AFHSB,2016[20] | 3 | 2 | 2 | 0 | 0 | 2 | 0 | 0 | 2 | 3 | 0 | 2 | 16/36 | 44.4 |
| Moore et al, 2016[42] | 3 | 3 | 3 | 2 | 3 | 2 | 1 | 3 | 3 | 3 | 2 | 0 | 28/36 | 77.8 |
| Stacey *et al* 2016[50] | 3 | 3 | 3 | 3 | 3 | 3 | 2 | 3 | 3 | 3 | 3 | 3 | 35/36 | 97.2 |
| AFHSB, 2017[21] | 3 | 2 | 2 | 0 | 0 | 2 | 0 | 0 | 2 | 3 | 0 | 2 | 16/36 | 44.4 |
| Deshwal *et al* 2017 [32] | 3 | 2 | 2 | 2 | 2 | 2 | 0 | 2 | 2 | 3 | 0 | 2 | 22/36 | 61.1 |
| Nelson *et al* 2017[43] | 3 | 3 | 3 | 3 | 3 | 3 | 2 | 3 | 3 | 3 | 3 | 3 | 35/36 | 97.2 |
| AFHSB, 2018[22] | 3 | 2 | 2 | 0 | 0 | 2 | 0 | 0 | 2 | 3 | 0 | 2 | 16/36 | 44.4 |
| Nelson *et al* 2018[44] | 3 | 3 | 3 | 3 | 3 | 3 | 2 | 3 | 3 | 3 | 3 | 3 | 35/36 | 97.2 |
| Nutong *et al* 2018[45] | 3 | 3 | 3 | 3 | 3 | 3 | 2 | 3 | 3 | 3 | 3 | 3 | 35/36 | 97.2 |
| Singer *et al* 2018[47] | 3 | 3 | 3 | 3 | 3 | 3 | 2 | 3 | 3 | 3 | 3 | 3 | 35/36 | 97.2 |
| AFHSB, 2019[23] | 3 | 2 | 2 | 0 | 0 | 2 | 0 | 0 | 2 | 3 | 0 | 2 | 16/36 | 44.4 |
| Barnes *et al* 2019[26] | 3 | 2 | 2 | 0 | 0 | 2 | 0 | 0 | 2 | 3 | 3 | 2 | 19/36 | 52.8 |
| King *et al* 2019 [40] | 3 | 3 | 3 | 3 | 3 | 3 | 3 | 2 | 3 | 3 | 3 | 3 | 35/36 | 97.2 |
| Donham *et al*, 2020 [34] | 3 | 3 | 3 | 3 | 3 | 3 | 3 | 3 | 3 | 3 | 3 | 3 | 36/36 | 100 |
| Gardner *et al* 2020 [36] | 3 | 3 | 3 | 3 | 3 | 3 | 3 | 3 | 3 | 3 | 3 | 3 | 36/36 | 100 |
| Ward *et al*, 2020 [54] | 3 | 3 | 3 | 3 | 3 | 3 | 3 | 3 | 3 | 3 | 3 | 3 | 36/36 | 100 |

| **QATSDD Criteria[15]** | | |
| --- | --- | --- |
| (1) Theoretical framework; | (6) Procedure for data collection | (11) Good justification for analytical method selected |
| (2) Aims/objectives; | (7) Rationale for choice of data collection tool(s) | (12) Strengths and limitations. |
| (3) Description of research setting | (8) Detailed recruitment data |  |
| (4) Sample size; | (9) Fit between research question and method of data collection (Quantitative only) |  |
| (5) Representative sample of target group | (10) Fit between research question and method of analysis (Quantitative only) |  |
| *QATSDD rating scale: 0=not at all; 1=very slightly; 2=moderately; 3=complete; AFHSB =*  *Armed Forces Health Surveillance Branch* | | |
